# Deimmunization of flagellin for repeated administration as a vaccine adjuvant

**DOI:** 10.1038/s41541-021-00379-4

**Published:** 2021-09-13

**Authors:** Koemchhoy Khim, Yong Jun Bang, Sao Puth, Yoonjoo Choi, Youn Suhk Lee, Kwangjoon Jeong, Shee Eun Lee, Joon Haeng Rhee

**Affiliations:** 1 Clinical Vaccine R&D Center, Chonnam National University, Hwasun-gun, Jeonnam Republic of Korea; 2 Combinatorial Tumor Immunotherapy MRC, Chonnam National University Medical School, Hwasun-gun, Jeonnam Republic of Korea; 3 Immunotherapy Innovation Center, Hwasun-gun, Jeonnam Republic of Korea; 4 Department of Microbiology, Chonnam National University Medical School, Hwasun-gun, Jeonnam Republic of Korea; 5 Vaxcell-Bio Therapeutics, Hwasun-gun, Jeonnam Republic of Korea; 6 Department of Pharmacology and Dental Therapeutics, School of Dentistry, Chonnam National University, Gwangju, Republic of Korea

**Keywords:** Adjuvants, Translational research

## Abstract

Flagellin, a protein-based Toll-like receptor agonist, is a versatile adjuvant applicable to wide spectrum of vaccines and immunotherapies. Given reiterated treatments of immunogenic biopharmaceuticals should lead to antibody responses precluding repeated administration, the development of flagellin not inducing specific antibodies would greatly expand the chances of clinical applications. Here we computationally identified immunogenic regions in *Vibrio vulnificus* flagellin B and deimmunized by simply removing a B cell epitope region. The recombinant deimmunized FlaB (dFlaB) maintains stable TLR5-stimulating activity. Multiple immunization of dFlaB does not induce FlaB-specific B cell responses in mice. Intranasally co-administered dFlaB with influenza vaccine enhanced strong Ag-specific immune responses in both systemic and mucosal compartments devoid of FlaB-specific Ab production. Notably, dFlaB showed better protective immune responses against lethal viral challenge compared with wild type FlaB. The deimmunizing B cell epitope deletion did not compromise stability and adjuvanticity, while suppressing unwanted antibody responses that may negatively affected vaccine antigen-directed immune responses in repeated vaccinations. We explain the underlying mechanism of deimmunization by employing molecular dynamics analysis.

## Introduction

Flagellin is a representative protein-based pathogen-associated molecular pattern (PAMP) cognitively recognized by Toll-like receptor-5 (TLR5) [1, 2]. Recognition of TLRs with cognate agonists activates innate immune responses and subsequently modulates adaptive immunity. TLR ligands are incorporated into vaccine formulations as adjuvants to effectively induce potent protective immune responses against co-formulated vaccine antigens [3]. Recently, it has been suggested that nonspecific activation of innate immune system manifests adaptive characteristics sufficient to protect host from later coming infections such as COVID-19, articulating a new jargon ‘trained innate immunity’ [4–6]. In this regard, pattern recognition receptor (PRR)-targeting vaccine adjuvants may serve dual players that enhance antigen-specific immune responses and train host innate immunity at the same time. We previously reported that bacterial flagellin, *Vibrio vulnificus* FlaB, is a versatile adjuvant applicable to wide spectrum of vaccines and immunotherapies [7–10]. When FlaB was administered with antigens (Ags) as mixture formulation or as a built-in adjuvant, FlaB strongly induced Ag-specific protective immune responses. The intranasally administered flagellin does not accumulate in olfactory nerve and bulb, guaranteeing no uptake into the central nervous system [11]. We also reported that FlaB-secreting *Salmonella typhimurium* effectively suppressed tumor growth and metastasis in mouse cancer models and prolonged survival through converting the tumor microenvironment towards tumor-suppressive condition [7]. In addition, flagellin-influenza vaccines have been tested in phase I/II clinical trials [12, 13], suggesting potent efficacy and safety profiles of flagellin in human applications. Given that flagellin is not only a strong immune modulator but also an immunogen in itself, in vivo administered flagellin adjuvant is likely to induce flagellin-specific immune responses. When vaccine administration is repeated, flagellin component may induce B-cell activation and antibody (Ab) production, interfering with the functions of subsequently administered flagellin-adjuvanted vaccines or immunotherapeutics and causing unwanted reactogenic responses [14]. Therefore, the development of flagellin derivatives not inducing flagellin-specific antibody without compromising the adjuvant activity would expedite clinical application.

In the present study, we hypothesized that deletion of B-cell epitopes in FlaB would restrain host antibody responses induced by repeated administration, which may make flagellins readily applicable to clinical grade vaccines and immunotherapeutics. Modifying or deleting appropriate amino acid sequences or domains without compromising the stable structure and TLR5 stimulating activity is pivotal in developing a deimmunized FlaB adjuvant. It was reported that *Salmonella typhimurium* FliC flagellin is comprised of four domains (D0, D1, D2, and D3) and TLR5-binding site is located at the D1 domain. Flagellin monomers are synthesized in the cytoplasm of the flagellated bacteria and transported to the cytoplasmic membrane to spontaneously polymerize to filamentous flagellum structure on the bacterial surface. The conserved TLR5-recognized short sequence in the D1 domain is buried inside when flagellar filament structure is formed, suggesting that monomeric flagellin released from the filament, but not the polymeric filamentous molecule, stimulates TLR5 [15]. The helical D0 and D1 domains are relatively well conserved while D2 and D3 domains are variable among different flagellated bacteria across genus and species. The D2 and D3 domains are exposed outward and induce specific antibody responses [16, 17]. *V. vulnificus*, a halophilic pathogenic bacterium, possesses a total of six flagellin genes and among the six flagellin genes, *flaB* is the major subunit contributing to the flagellum biogenesis and the function, indicating FlaB should have been conserved physico-chemically stable throughout the long history of natural evolution [18]. Here, we employed computational prediction for B-cell epitopes to identify immunogenic determinants inducing specific antibody responses in FlaB hypervariable D2-D3 domains. We generated a D2D3 domain-depleted FlaB (FlaB^ΔD2D3^) and a truncated variant (dFlaB) based on the in silico prediction. The freshly purified recombinant dFlaB, a less self-polymerizing mutant protein, induced stable TLR5-stimulating activity. However, the FlaB^ΔD2D3^ protein appeared unstable, resulting in compromised TLR5-stimulating activity under environmental challenges.

Here, we report a deimmunized stable flagellin (dFlaB) having comparable TLR5 stimulating potency and significant therapeutic benefit of the dFlaB as an immunomodulator. We show that multiple immunization of the dFlaB does not induce FlaB-specific Ab responses using mouse immunization models. When mucosal adjuvant activity of the flagellins was assessed, comparable levels of adjuvant activity was observed in both dFlaB and wild type (WT) FlaB. To presume dFlaB’s clinical benefits, we employed a lethal influenza virus challenge experiment. Notably, three-time vaccination with dFlaB-adjuvanted H1N1 mucosal vaccine induced significantly stronger protection against lethal virus challenge compared with FlaB plus H1N1 vaccine. It was interesting that the survival was significantly higher in dFlaB-adjuvanted vaccinee animals while induced antibody titers and antiserum neutralizing activities were comparable or lower than with WT FlaB-adjuvanted vaccinees, respectively, suggesting antibody-noninducing dFlaB’s additional advantages.

## Results

### Development of deimmunized FlaB by deleting B-cell epitope in the variable region of FlaB

**Fig. 1:**
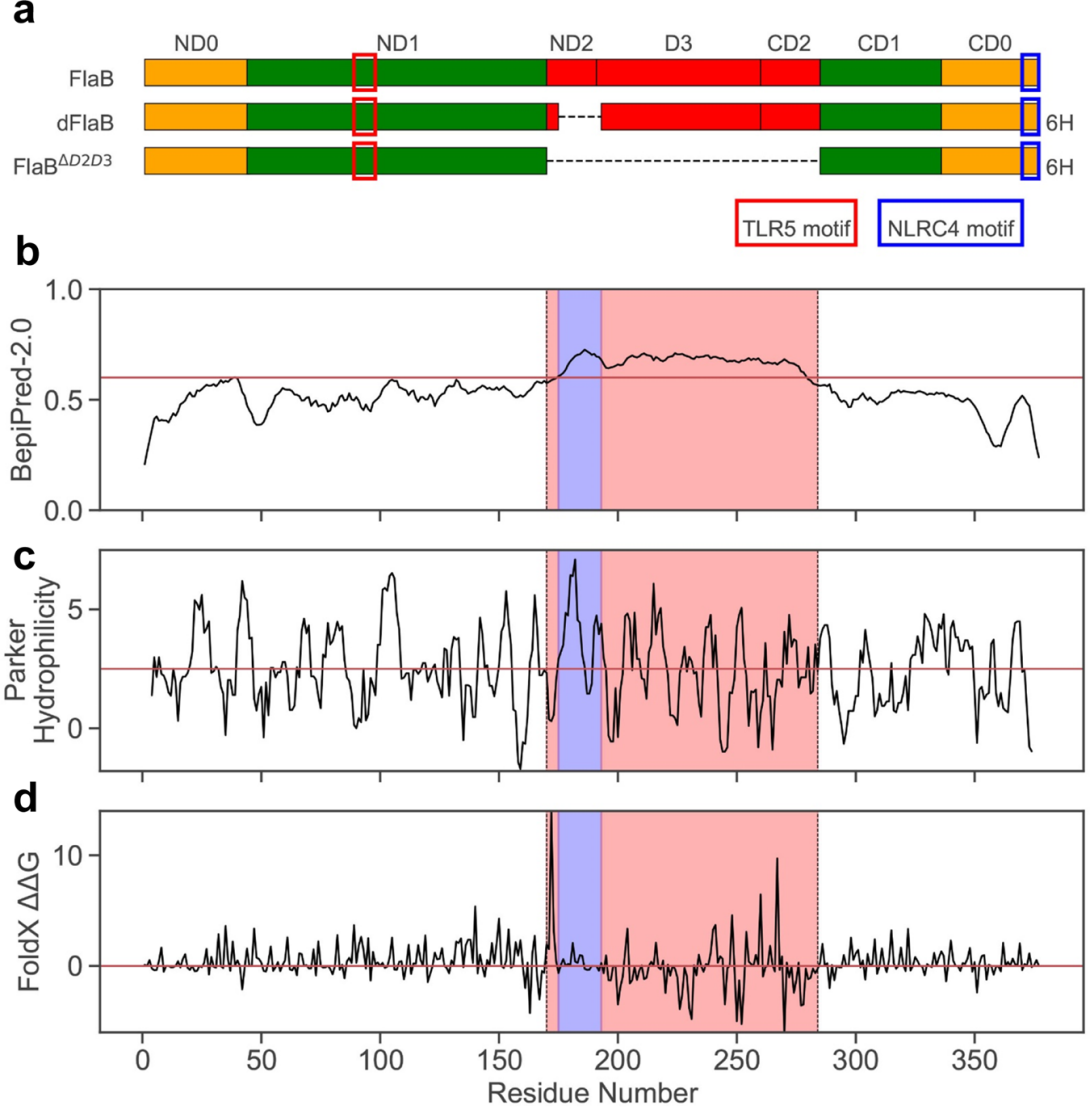
Development of deimmunized flagellins. **a** Schematic view of deimmunized FlaB variants. Wild type *Vibrio vulnificus* FlaB consists of conserved N- and C-terminal domains required for flagellum biogenesis. dFlaB and FlaB^ΔD2D3^ were deimmunized by deleting potential B-cell epitopes in ND2, D3, and CD2, where variability among different bacterial genus and species. **b** BepiPred-2.0 predicted ND2 to CD2 domains being most immunogenic. **c** The Parker hydrophilicity predicted that the most of ND2 domain may also be immunogenic. **d** In silico alanine scanning by FoldX additionally identified residues in little interactions with other regions (near zero). Based on these three metrics, we selected a linear region of 19 amino acids (175 SYQAEEGKDKNWNVAAGDN 193; shaded in blue) for deimmunization. The red box in ND1 represents TLR5 binding motif while blue box in CD0 represents NLRC4 binding motif, respectively. The 6H represented 6xHis-Tag for purification of recombinant dFlaB and FlaB^ΔD2D3^.

Flagellin is a strong immune modulator that enhances specific immune responses against co-administered Ags and easily engineered with protein antigens as built-in adjuvants [10, 19]. Since flagellin is well documented as an immunogenic protein being used as a vaccine antigen or a diagnostic target for previous infections, flagellin-specific Ab production is a major concern for repeated administration as adjuvant or immunotherapeutic. In order to develop a druggable flagellin, we attempted deimmunization of *V. vulnificus* FlaB that our group has been studying as an adjuvant for various vaccine antigens. Firstly, potential B-cell epitopes of FlaB were predicted using BepiPred-2.0 with the threshold value > 0.6 [20]. The variable domain 2 and 3 (ND2-D3-CD2) were predicted to harbor dominant B-cell epitopes (Fig. 1A, B). As most B-cell epitopes are known to be conformational [21], to verify the predicted B-cell epitopes, we built a homology model of FlaB using MODELLER [22] with two most homologous structure templates (PDB ID 3K8V and 6JY0). Homology models were further energy-minimized using the TINKER [23] molecular dynamics package (AMBER99sb) [24] with the GB/SA implicit solvent model [25]. The model monomers were assembled and arranged onto a complex structure of *S. Typhimurium* flagellin (PDB ID: 6JY0). Structural analyses show that the epitope region in D2 and D3 are solvent-exposed in both complex and monomeric forms (Supplementary Fig. 1). We thus removed entire D2 and D3 from FlaB for deimmunization (FlaB^ΔD2D3^). In addition to the ΔD2D3 variant, we also identified a specific region (175 SYQAEEGKDKNWNVAAGDN 193) in ND2 and D3 that has relatively higher epitope score as well as the Parker hydrophilicity metric (Fig. 1c) [26] but the lowest energy impacts according an in silico alanine scanning using FoldX (Fig. 1d) [27]. In order to develop deimmunized flagellin, from which only an essential minimum of amino acids were removed, we constructed a flagellin variant only lacking above the 19 amino acids (dFlaB).

**Fig. 2:**
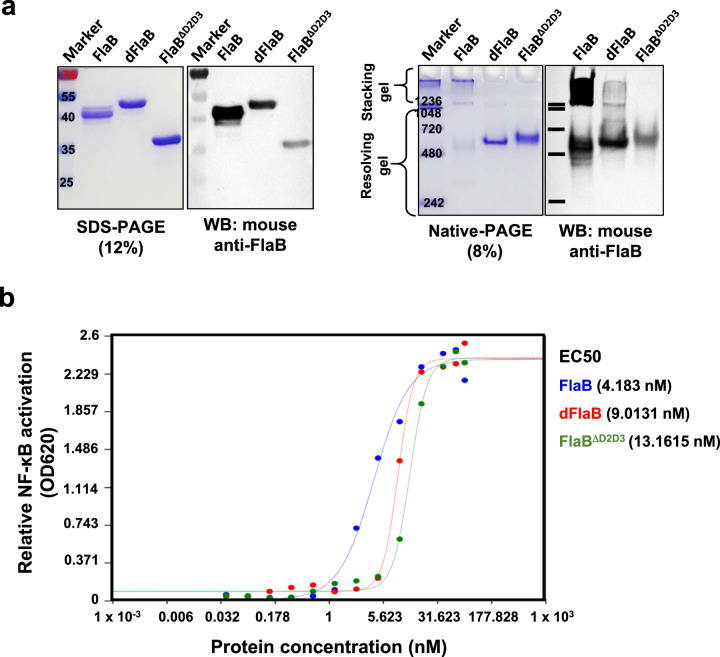
Characteristics of the recombinant proteins. **a** Characterization of dFlaB and FlaB^ΔD2D3^ by sodium dodecyl sulfate polyacrylamide gel electrophoresis (SDS-PAGE), native-PAGE, and subsequent Western blot analysis with mouse anti-FlaB serum induced by intraperitoneal immunization of FlaB formulated complete Freund’s adjuvant. **b** Determination of TLR5-dependent NF-κB stimulating activity by FlaB, dFlaB, and FlaB^ΔD2D3^. The relative NF-κB activities were analyzed by using HEK-Blue^TM^ hTLR5 cells and HEK-Blue^TM^ detection assay systems. EC50 were calculated using triplicate OD 620 nm values for each protein concentration over a wide range of protein concentrations (0.03719 nM to 76.16 nM at the AAT Bioquest website (https://www.aatbio.com/tools/ec50-calculator). The same molar ratio of proteins was used, and PBS was used as a negative control.

We constructed expression plasmids to produce recombinant dFlaB and FlaB^ΔD2D3^ proteins (Table 1). The DNA sequences of the resulting expression vectors were confirmed by the dideoxy-chain termination sequencing method via the Macrogen Online Sequencing Order System (http://dna.macrogen.com/kor/) (Supplementary Fig. 2). The recombinant proteins were purified by affinity chromatography and confirmed by SDS-PAGE, native-PAGE, and Western blot analysis using FlaB-specific mouse antiserum, which was raised by subcutaneous immunization as a mixture with complete Freund’s adjuvant. Despite estimated molecular mass of dFlaB (40.7 kDa) is smaller than the wild type FlaB (41.47 kDa), dFlaB showed slower mobility on SDS-PAGE than wild type FlaB (Fig. 2a). We confirmed that the recombinant dFaB mutant was correctly expressed by MALDI-TOF analysis (Supplementary Fig. 3). When the same amount of protein is loaded, dFlaB and FlaB^ΔD2D3^ showed fainter band intensity in both Western blot analyses following SDS-PAGE and native-PAGE (Fig. 2a). The results suggest that FlaB D2 and D3 domains are more antigenic and 19 amino acids (175~193) play a dominant role in inducing FlaB-specific Ab response. To test TLR5-stimulating activities of the proteins, we measured TLR5-dependent NF-κB signaling by using freshly purified proteins immediately after the solvent change with phosphate buffered saline (PBS). As shown in Fig. 2b, the fresh dFlaB and FlaB^ΔD2D3^ induced TLR5-stimulating activity in a dose dependent manner. Half maximal effective concentration (EC50) of FlaB, dFlaB, or FlaB ^ΔD2D3^ was calculated using triplicate OD 620 values for each protein concentration over a wide range of protein concentrations (0.03719 nM to 76.16 nM). The calculated EC50 values were 4.183 nM for FlaB, 9.0131 nM for dFlaB, and 13.1615 nM FlaB^ΔD2D3^. This result indicates that dFlaB and FlaB^ΔD2D3^ display slightly decreased activity compared to FlaB. Notably, in higher than 9.52 nM of protein concentrations, TLR5-dependent NF-kB activation showed no significant difference between FlaB and dFlaB (*P* = 0.2064). The native-PAGE data revealed that freshly purified dFlaB and FlaB^ΔD2D3^ proteins in PBS showed lower polymerization tendency than WT FlaB. When the recombinant proteins were stored long-term under various environmental conditions, FlaB^ΔD2D3^ protein was subject to aggregation resulting in compromised TLR5-stimulating activity (Supplementary Fig. 4). Collectively, these results show that dFlaB, where the most immunogenic 19 amino acid B cell epitope was deleted, maintains physicochemical stability and TLR5-stimulating activity.

**Table 1:** Bacterial strains, plasmids, and influenza virus.

	**Description**	**Source**
Bacteria		
*Escherichia coli* DH5α	F ^− ^*recA1* restriction negative	Laboratory collection
*E. coli* ER2566	F^−^ λ^−^ *fhuA2 [lon] ompT lacZ::T7 gene1 gal sulA11∆(mcrC-mrr)114::IS10 R(mcr-73::miniTn10-TetS)2* *R(zgb-210::Tn10)(*TetS*) endA1 [dcm]*	New England Biolabs, Inc.
*E. coli* BL 21 (DE3)	*hsdS gal (λcIts857 ind1 Sam7 nin5 lacUV5-T7 gene1)*	Laboratory collection
Plasmid		
pCR2.1TOPO	Cloning vector; Ap^r^; Km^r^	Invitrogen
pTYB12	N-terminal fusion expression vector in which the N terminus of a target protein is a fused Intein-tag; Ap^r^	New England Biolabs, Inc.
pET30a+	N-terminal fusion expression vector in which the N terminus of a target protein is a fused His-tag; Km^r^	EMD Bioscience
pCMM250	1.5-kb *EcoRI-PstI* fragment containing ORF of flaB cloned into pTYB12	[28]
pET-30a(+)::dFlaB	pET-30a(+) plasmid containing a DNA-fragment of FlaB with deletion of spanned 175–193 amino acid at *NdeI*-*XhoI*	This study
pET-30a(+)::FlaB^ΔD2D3^	pET-30a(+) plasmid containing a DNA-fragment of FlaB with deletion of D2D3 region at *NdeI*-*XhoI*	This study
		This study
Influenza virus		
H1N1	A/Brisbane/59/2007	Centers for Disease Control and Prevention [11, 50]

### Repeated immunization of dFlaB does not induce flagellin-specific Abs

**Fig. 3:**
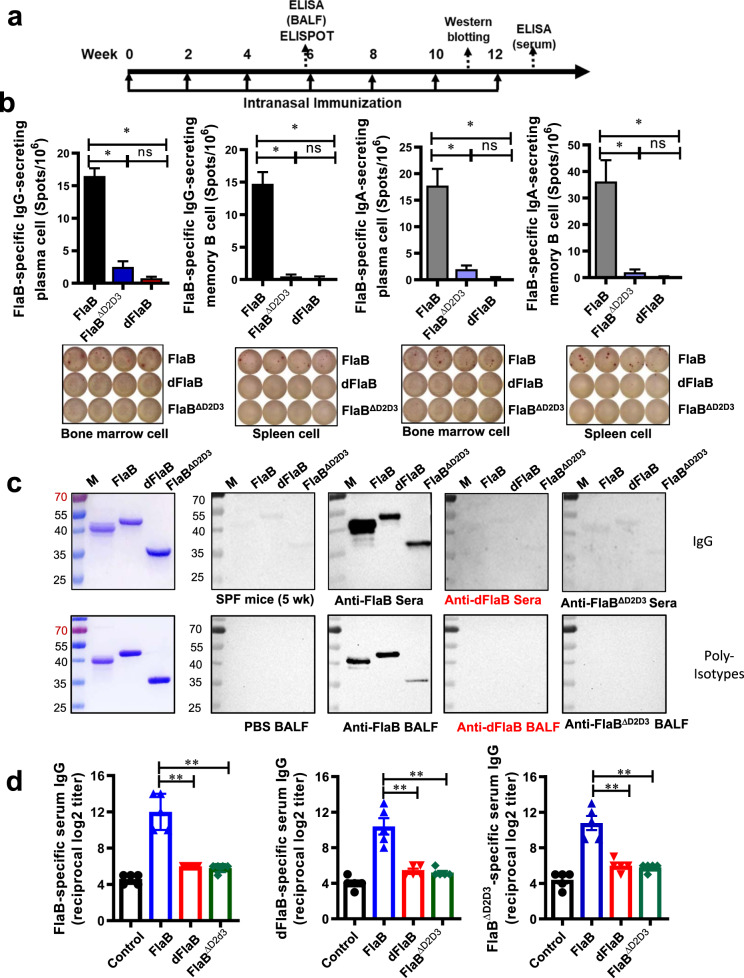
Multiple immunization of dFlaB does not induce FlaB-specific Ab responses. **a** Experimental schedule of multiple immunization with FlaB, dFlaB or FlaB^ΔD2D3^. Groups of BALB/c mice (*n* = 5) were intranasally immunized with phosphate buffered saline (PBS), 4 μg FlaB, 4 μg dFlaB and 2.84 μg FlaB^ΔD2D3^ seven times in 2-week intervals, and FlaB-specific B cell responses were determined by enzyme-linked immune absorbent spot (ELISPOT) assay, Western blot analyses and enzyme-linked immunosorbent assay (ELISA). **b** FlaB-specific memory B and plasma cell responses induced by multiple immunization of 4 μg FlaB, 4 μg dFlaB and 2.84 μg FlaB^ΔD2D3^ ELISPOT assay. Two weeks after the 3^rd^ immunization, FlaB-specific IgG- and IgA-secreting plasma cells from bone morrow and IgG- and IgA-secreting memory B cells from spleen were determined, respectively. **c** FlaB-specific Ab production evaluated by Western blot analysis. One week after the 6th immunization, anti-sera (anti-FlaB, anti-dFlaB, and anti-FlaB^ΔD2D3^) were prepared from the immunized mice. Two weeks after three-time immunization, BALF was prepared from the immunized mice. Purified FlaB, dFlaB, or FlaB^ΔD2D3^ were run by SDS-PAGE and probed by Western blot analysis with the anti-sera or BLAF. **d** Flagellin-specific Ab titers after seven immunizations. One week after the last immunization, sera were collected and FlaB-, dFlaB- or FlaB^ΔD2D3^-specific IgG titers were determined by ELISA. Mann–Whitney test was used to compare two groups. *P < 0.05; **P < 0.01; and ***P < 0.001; ns, non-significant.

Next, to test whether the engineered dFlaB protein does not induce FlaB-specific immune responses in vivo, we compared antibody responses induced by repeated immunization with FlaB, dFlaB, or FlaB^ΔD2D3^ proteins. Groups of BALB/c mice were intranasally vaccinated with flagellin proteins seven times at 2-week intervals (Fig. 3a) and the FlaB-specific B cell responses were determined at appropriate time points. The reason why intranasal route was employed is that we have observed, in previous studies, that flagellin serves an excellent mucosal vaccine adjuvant inducing efficacious immune responses in both mucosal and systemic compartments [10, 11, 28]. After three times immunization, FlaB-specific IgG-secreting plasma cells and memory B cells were assayed by ELISPOT [29]. As shown in Fig. 3b, FlaB, FlaB^ΔD2D3^or dFlaB produced 16.50 ± 1.19, 2.50 ± 0.87, or 0.75 ± 0.25 FlaB-specific IgG secreting plasma cells in bone marrow and 14.75 ± 1.80, 0.50 ± 0.29, or 0.25 ± 0.25 memory B cells in spleen, respectively. Given that mucosal secretory IgA (SIgA) has potent protective efficacy at mucosal infection sites, we also determined FlaB-specific IgA secreting plasma and memory B cells. Three times immunization with FlaB, FlaB^ΔD2D3^or dFlaB produced 17.75 ± 3.63, 1.41 ± 0.82, or 0.50 ± 0.29 FlaB-specific IgA secreting plasma cells in bone marrow and 36.25 ± 9.25, 2.00 ± 1.25, or 0.25 ± 0.29 memory B cells in spleen, respectively. To confirm whether even multiple immunization of the dFlaB still does not induce FlaB-specific Ab, we continued seven times immunization with FlaB, dFlaB, or FlaB^ΔD2D3^ proteins. After six times immunization, we compared antigen-recognition patterns of the FlaB-, dFlaB- or FlaB^ΔD2D3^-elicited anti-sera by Western blotting. To determine Ab production in mucosal secretions, two-weeks after the third vaccination, we collected BALF and determined FlaB-specific Ab responses by Western blot analysis. Since it has been reported that deletions of hypervariable region of *Salmonella typhimurium* FliC abrogates antibody-mediated neutralization [16, 17], we employed FlaB^ΔD2D3^ as a control protein to investigate the role of 19 amino acids on the Ab production as well as Ag–Ab interaction profiles. As shown in Fig. 3c, anti-FlaB serum and BALF recognized corresponding FlaB, dFlaB, and FlaB^ΔD2D3^ proteins. Though we loaded same amount of proteins on the polyacrylamide gel, the Western blot showed stronger band intensity for WT FlaB protein compared with dFlaB and FlaB^ΔD2D3^ proteins, suggesting the variable D2 and D3 domains dominantly induce specific antibodies. The anti-dFlaB and anti-FlaB^ΔD2D3^ sera barely detected FlaB-, dFlaB- or FlaB^ΔD2D3^ proteins. The anti-FlaB^ΔD2D3^ sera showed similar pattern with that recognized by normal naive sera. After the multiple immunization, we determined FlaB-, dFlaB- or FlaB^ΔD2D3^-specific serum IgG titers in anti-FlaB, anti-dFlaB, and anti-FlaB^ΔD2D3^-sera. Seven-time immunization of WT FlaB induced significant levels of FlaB-, dFlaB, and FlaB^ΔD2D3^-specific serum Log2 IgG titers (12.00 ± 0.89, 10.4 ± 0.93, and 10.8 ± 0.80 respectively). While dFlaB and FlaB^ΔD2D3^ immunization resulted in 6.0 ± 0.0 and 5.8 ± 0.2 FlaB-specific serum IgG titers, respectively, indicating dFlaB and FlaB^ΔD2D3^ were significantly attenuated in inducing FlaB-specific serum IgG even after seven-time repeated intranasal immunizations (Fig. 3d). These results collectively indicate that the 19 amino acid hydrophilic domain serves an essential B cell epitope as well as where anti-FlaB Abs dominantly bind. Furthermore, the deletion of only 19 amino acid is sufficient to inhibit FlaB-specific Ab production while the stability is not compromised.

### Biodistribution of in vivo*-*administered FlaB or dFlaB

**Fig. 4:**
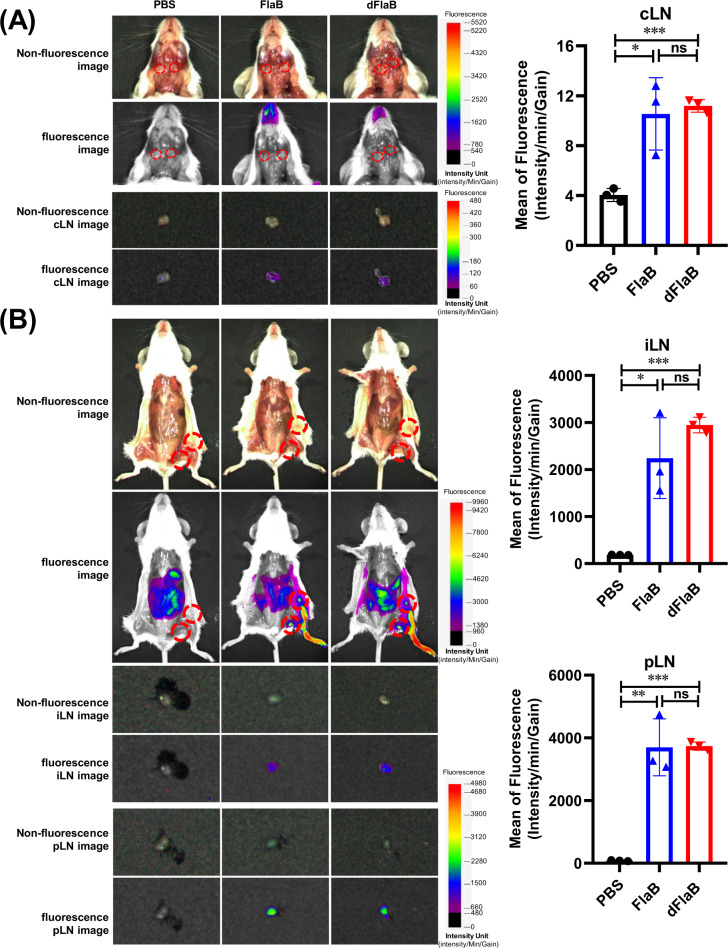
Biodistribution of in vivo administered flagellins. **a** Biodistribution of intranasally administered FlaB or dFlaB. BALB/c mice were intranasally administered with 100 μg of FNR675-conjugated FlaB or dFlaB. **b** Biodistribution of FlaB or dFlaB administered by footpad injection. BALB/c mice were administered with 100 μg of FNR675-conjugated FlaB or dFlaB. Six hours after the administration, the cLNs, iLNs, and pLNs were isolated to determine the biodistribution of the FlaB or dFlaB by measuring fluorescence intensity as described in Materials and methods.

To visualize in vivo-administered FlaB or dFlaB, we generated fluorescence-conjugated proteins [8] and then the biodistribution was determined by measuring fluorescence signals in the draining lymph nodes. Purified FlaB or dFlaB was mixed with FNR675-NHS ester (BioActs, Korea) at 4 °C and maintained overnight in the dark with stirring. The labeled FlaB- or dFlaB-FNR675 was then separated from the unconjugated dye using a centrifugal filter (10 kDa cutoff) (Amicon Ultra®-4, UFC801024), followed by washing (5x) in PBS. Next, the amount of conjugated protein was determined from the calibration curve of the FNR675-NHS ester using a UV-Vis spectrophotometer (UV-2700, Shimadzu, Japan). BALB/c mice were administered with 100 μg of FlaB-FNR675 or dFlaB-FNR675 through intranasal or footpad route. Six hours after intranasal administration, the cervical lymph node (cLN) was isolated to measure fluorescent photons. Significant fluorescence signal was detected in both FlaB- or dFlaB-administered mice compared to PBS-administered group with no significant differences between two test groups (*P* < 0.05 for PBS vs FlaB; *P* < 0.001 for PBS vs dFlaB; *P* > 0.05 for FlaB vs dFlaB) (Fig. 4a). We also determined biodistribution of FlaB or dFlaB in inguinal lymph node (iLN) and popliteal lymph node (pLN) after footpad injection. As shown in Fig. 4b, both FlaB and dFlaB localized in the draining iLN and pLN with comparable levels in fluorescence intensity (*P* > 0.05 for FlaB vs dFaB). These results clearly indicate that in vivo-administered dFlaB similarly distributed in the dLNs as compared with WT FlaB.

### dFlaB-adjuvant exert equal efficacy to WT FlaB in inducing influenza vaccine-specific Ab responses

Next, to confirm whether the dFlaB maintains equivalent mucosal adjuvant activity as WT FlaB, we performed in vivo immunization experiments employing a commercial influenza vaccine. We compared Ag- and FlaB-specific Ab responses in the same way described above. Previously we had shown that FlaB-adjuvanted inactivated influenza vaccine induced excellent protective immunity in both mucosal and systemic compartments [11]. It has been reported that influenza A H3N2 infection results in severer morbidity than influenza H1N1 or type B infection [30, 31]. All influenza vaccines for 2019–2020 season contain two influenza A viruses (H1N1 and H3N2) and one or two influenza B virus. Observational studies have consistently shown that influenza vaccine effectiveness is lower for H3N2 relative to H1N1pdm09 and type B, and this is not entirely explained by antigenic match [32]. In the present study, we compared adjuvant activities of dFlaB and FlaB by co-administering inactivated H3N2 A/Switzerland/9715293/2013 NIB-88 split vaccine (sH3N2; IL-YANG PHARM. Yongin, Korea). To evaluate Ag-specific Ab responses induced by the mucosal immunization of dFlaB-adjuvanted H3N2 split vaccine (sH3N2), we measured H3N2-specific IgG and IgA titers in blood and BALF, respectively. BALB/c mice were intranasally vaccinated three 3 times with PBS (PBS), 4 μg dFlaB (dFlaB), 4 μg FlaB (FlaB), 1.5 μg sH3N2 (sH3N2), 1.5 μg sH3N2 plus 4 μg dFlaB (sH3N2 + dFlaB), and 1.5 μg sH3N2 plus 4 μg dFlaB (sH3N2 + FlaB) at two-week intervals. Two weeks after the last immunization, the sH3N2- or FlaB-specific Ab responses in systemic (serum) and mucosal (BALF) compartments were assayed by ELISA and Western blot analysis.

**Fig. 5:**
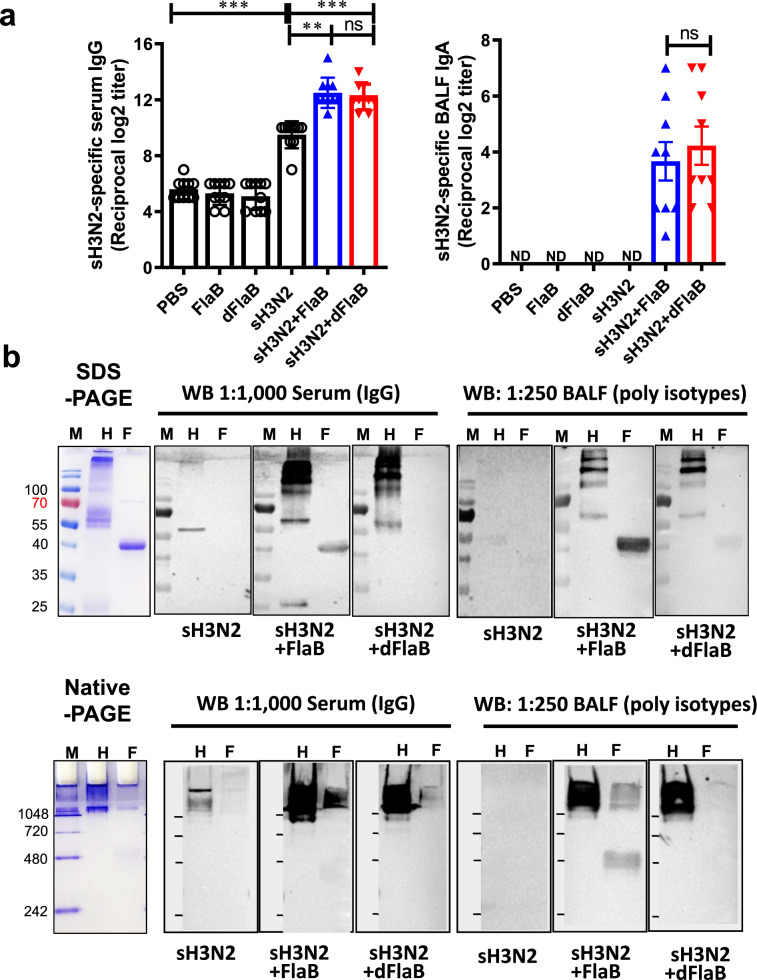
The dFlaB has strong mucosal adjuvant activity without FlaB-specific Ab induction. **a** Ag-specific antibody response after intranasal vaccination. Groups (*n* = 9–10) of mice were intranasally vaccinated with PBS (PBS), 4 μg FlaB (FlaB), 4 μg dFlaB (dFlaB), 1.5 μg H3N2 A/Switzerland/9715293/2013 NIB-88 split vaccine (sH3N2), 1.5 μg sH3N2 plus 4 μg FlaB (sH3N2 + FlaB), or 1.5 μg sH3N2 plus 4 μg dFlaB (sH3N2 + dFlaB) three times at two-week intervals. Two weeks after the last immunization, serum or BALF was collected and sH3N2-specific serum IgG or BALF IgA titers were determined by ELISA. **b** Determination of sH3N2- or FlaB-specific Ab production. Two weeks after the last immunization, serum or BALF was collected. SDS-denatured or non-denatured sH3N2 or FlaB was resolved by PAGE. The sH3N2- or FlaB- specific Abs in the sera and BALF were probed by Western blotting analysis. Mann-Whitney test was used to compare two groups. ***P < 0.001; ns, non-significant; ND, not detected (under the detection limit); M, marker; H, sH3N2; F, FlaB.

Co-administration of FlaB or dFaB in combination with sH3N2 Ag induced significantly higher levels of anti-sH3N2 serum IgG (*P* < 0.01 for sH3N2 vs sH3N2 + FlaB; *P* < 0.001 for sH3N2 vs sH3N2 + dFlaB). The sH3N2-specific IgA was detected in the FlaB- or dFlaB-adjuvanted vaccination only, suggesting secretory IgA production is dependent on the mucosal adjuvant. The dFlaB exerted equivalent adjuvant activities with WT FlaB in both systemic IgG and mucosal IgA levels (*P* > 0.05 for sH3Ns + FlaB vs sH3Ns + dFlaB) (Fig. 5a and Supplementary Fig. 5). To ascertain the ELISA result, we performed Western blot analyses following SDS-PAGE or native-PAGE by using anti-sera and anti-BALF. As shown in Fig. 5b, the anti-sH3N2 sera detected both SDS-denatured and native sH3N2 Ags with weak binding intensity. Expectedly, the anti-dFlaB + sH3N2 or anti-FlaB + sH3N2 serum more strongly recognized both denatured and native H3N2 antigens than anti-sH3N2 serum under 1:1,000 serum dilution. Notably, in contrast to the anti-FlaB + sH3N2 serum recognizing both denatured and native form of FlaB, anti-dFlaB + sH3N2 serum could not detect denatured FlaB. The anti-dFlaB + sH3N2 serum minimally reacted with non-denatured form of FlaB. To confirm whether intranasal immunization efficiently induced corresponding mucosal immune responses, we also determined sH3N2- or FlaB-specific Ab responses by Western blot analysis using BALF. The anti-sH3N2 BALF barely detected both denatured and native sH3N2. The anti-dFlaB + sH3N2 or anti-FlaB + sH3N2 BALF strongly detected both denatured and native H3N2 antigens, indicating both dFlaB and FlaB have efficacious mucosal adjuvant activities generating protective secretory antibody responses. Compared with WT FlaB + sH3N2 vaccination, dFlaB-adjuvanted vaccine did not induce efficient FlaB-recognizing Igs in the BALF. Taken together, dFlaB maintains mucosal adjuvant activity without inducing FlaB-specific Ab responses in this influenza vaccination model, suggesting clinical applicability of dFlaB in future mucosal vaccines against various viral infections.

### dFlaB-adjuvanted vaccine confer enhanced protection against lethal influenza challenge

**Fig. 6:**
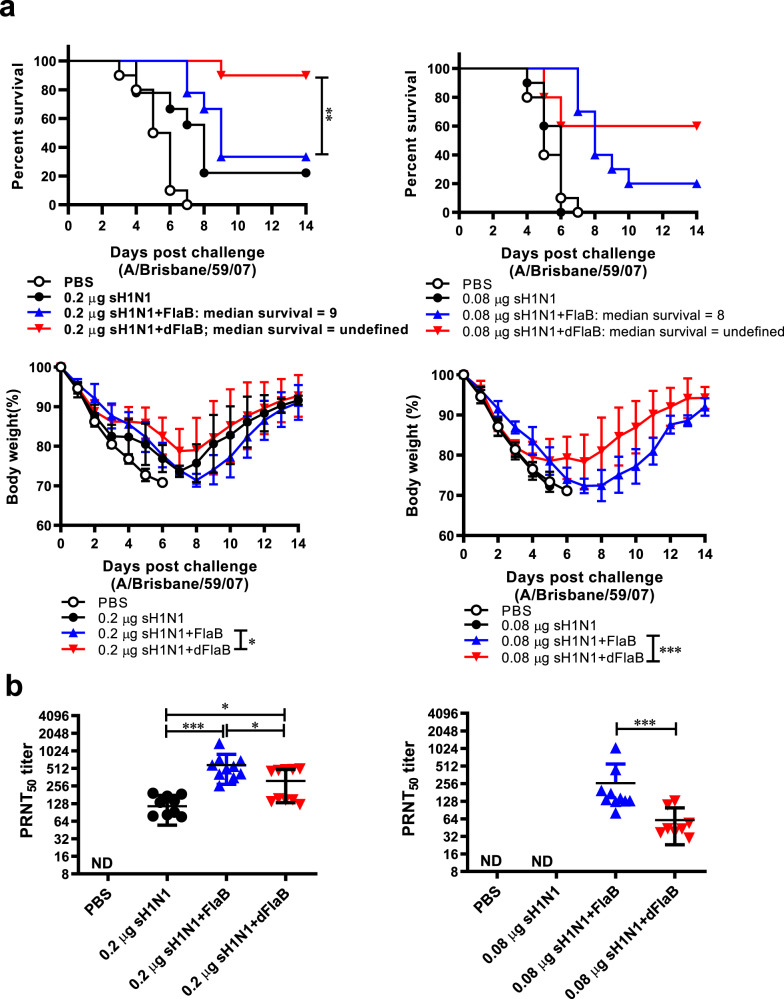
The dFlaB-adjuvant induced better protection than that of wild type FlaB in a H1N1 influenza lethal challenge model. Groups of mice (*n* = 9–10) were intranasally immunized with PBS, FlaB, dFlaB, H1N1 A/Brisbane/59/07 split vaccine (sH1N1), sH1N1 plus FlaB (sH1N1 + FlaB), or sH1N1 plus dFlaB (sH1N1 + dFlaB) three times at a two-week interval. Two weeks after the immunization, mice were intranasally challenged with a 2.4 × LD_50_ live A/Brisbane/59/07 influenza virus. After the challenge, survival rate and changes in body weight (**a**) were monitored for 2 weeks. Naive mice were used as controls for the determination of body weight change. **b** Plaque reduction neutralization test (PRNT) using live influenza A/Brisbane/59/07 virus. Two weeks after the last immunization, serum was collected from the retroorbital plexus. PRNT_50_ titer was determined as 50% neutralization of plaques based on positive control virus wells. Mann–Whitney test was used to compare two groups.

To comparatively assess the protective efficacies of the dFlaB- and FlaB-adjuvanted vaccines against lethal virus infection, we performed a challenge study using mouse-adpated virulent H1N1 A/Brisbane/59/07 influenza virus [11]. We tested two Ag doses to reproduce sufficient and suboptimal immunization conditions. BALB/c mice were intranasally vaccinated three times with PBS, inactivated H1N1 A/Brisbane/59/07 split vaccine (Green Cross, Hwasun, Korea; 0.2 μg for sufficient Ag dose or 0.08 μg for suboptimal Ag dose) plus 4 μg dFlaB (sH1N1 + dFlaB), and sH1N1 plus 4 μg FlaB at two-week intervals. Two weeks after the last immunization, serum samples were collected and vaccinated mice were challenged with lethal dose of live A/Brisbane/59/07 virus (2.4× LD_50_). Survival and body weight changes were monitored for 14 days. As shown in Fig. 6a, in both challenge experiments, all of the vehicle immunized mice (PBS) lost 30% of their body weight by day 7 with 0% survival. The final survival percentages of 0.2 μg sH1N1 + dFlaB or 0.2 μg sH1N1 + FlaB-vaccinated mice were 90% and 33.3%, respectively. Mice immunized with 0.2 μg sH1N1 + dFlaB showed a significantly increased survival rate compared to those immunized with sH1N1 + FlaB (*P* = 0.0094). In the suboptimal Ag dose (0.08 μg sH1N1) immunization setting, dFlaB-adjuvanted group showed 60% survival at day 14. However, 0.08 μg sH1N1 + FlaB group showed only 20% survival. Median survival period of the FlaB-adjuvanted sH1N1 vaccinated group (0.08 μg sH1N1 + FlaB) was 8 days. Both dFlaB-adjuvanted sH1N1-vaccinated groups showed significant reductions in morbidity as determined by body weight changes (*P* < 0.05 for 0.2 μg sH1N1 + dFlaB vs 0.2 μg sH1N1 + FlaB; *P* < 0.001 for 0.08 μg sH1N1 + dFlaB vs 0.08 μg sH1N1 + FlaB) (Fig. 6a). To test virus-neutralization capacity of anti-sera, we performed a virus plaque reduction neutralization test (PRNT). As shown in Fig. 6b, FlaB- or dFlaB-adjuvanted vaccine showed significantly higher titers than the 0.2 μg sH1N1 alone (*P* < 0.05 for 0.2 μg sH1N1 vs 0.2 μg sH1N1 + FlaB; *P* < 0.001 for 0.2 μg sH1N1 vs 0.2 μg sH1N1 + dFlaB). Specifically, the sH1N1 + dFlaB groups showed significantly lower PRNT levels compared with sH1N1 + FlaB, suggesting the dFlaB-mediated adjuvanticity would employ other components of immunity than virus-specific neutralizing antibody responses. We also measured Ag-specific serum IgG titers. As shown in Supplementary Fig. 6, similar level of H1N1-specific serum IgG was detected in FlaB- or dFlaB- adjuvanted group. When we compared the body weight change, the sH1N1 + dFlaB groups showed severe body weight loss in earlier time points (until day 3 for 0.2 μg sH1N1 and day 5 for 0.08 μg sH1N1 Ag doses). Three- or five-days after the virus challenge, groups of mice immunized with dFlaB-adjuvanted vaccine showed faster body weight recovery (Fig. 6a). This result clearly suggests that deimmunization potentiates immune modulatory function of flagellin against lethal infection and the dFlaB would have advantage over WT FlaB as a vaccine adjuvant with virtues other than antibody-noninducing characteristics. It will be interesting, in future studies, to check the ability of dFlaB in inducing trained innate immunity [4–6] or cell mediated immunity against viral antigens.

## Discussion

Flagellin, the major structural component of flagellum biogenesis [18], is a unique protein-based TLR agonist that serves as an efficacious adjuvant for various mucosal vaccines and immunotherapeutics [7–11, 19, 28]. Immune responses against protein therapeutics itself would be detrimental to efficacy and safety. Though it has been reported that there was no apparent relationship between anti-flagellin Ab and adverse events after influenza–flagellin fusion vaccine administration in a clinical trial [33], since flagellin is a strong immunogen [34] the flagellin-specific Ab production has continuously raised concern for the development of flagellin-based therapeutics. Repeated immunization with flagellin-adjuvanted vaccine induces high titer of flagellin-specific Ab production, which may interfere with subsequently administered flagellin-adjuvanted vaccine resulting in unwanted neutralizing or reactogenic responses. Therefore, deimmunization of flagellin that preserve adjuvant activity is a promising option to make a pharmaceutically optimized flagellin. Modification of proteins, being highly optimized throughout long history of evolution, often results in drastic deterioration in physicochemical stability. In the present study, we reported a rather conservative way of deimmunizing highly antigenic protein flagellin by deleting minimal number of amino acids constituting a dominant B-cell epitope.

In the present study, we generated a deimmunized version of flagellin (dFlaB). To develop deimmunized FlaB, we targeted B-cell epitopes by using *in silico* prediction and structure modeling tools. It has been reported that deletions of hypervariable region of *Salmonella typhimurium* FliC abrogates antibody-mediated neutralization [16]. Our computational analyses also found that the most probable B-cell epitope would be located within hypervariable D2D3 regions (170–284 amino acid) of the FlaB molecule. According to the 3D structure prediction, D2D3 deletion (FlaB^ΔD2D3^) do not influence TLR5-binding motif (89–96 amino acid). However, we noted that FlaB^ΔD2D3^ should have limited usefulness a pharmaceutical because of its poor stability in vitro (Supplementary Fig. 4) and in vivo. It has been reported that the D3 domain of *S. typhimurium* FliC play an important role in the stabilization of the D1 and D2 domains [35]. As shown in Figure 1, since the deleted 19 amino acids of dFlaB encompass 17 amino acids of ND2 and 2 amino acids of D3 domain, it is likely that the dFlaB preserve the essential amino acid sequences responsible for stabilization of the flagellin structure without disrupting folding kinetics. From the native-PAGE and Western blot analyses of dFlaB, we found that it tends to less polymerize in the PBS condition (Fig. 2b). It was reported that a short-conserved sequence buried in the polymeric flagella filament serves the TLR5 recognition site, which suggests that monomeric flagellin, but not the polymeric filamentous molecule, stimulates TLR5 [36]. As shown in Fig. 2a, dFlaB showed slow mobility on SDS-PAGE than WT FlaB, suggesting probable conformational change not compromising with TLR5 binding. In our previous report, we observed same molecular mass FlaB-PspA and PspA-FlaB fusion polypeptides showed significantly different mobility on SDS-PAGE gel [19]. Later, as will be discussed below, it was presumed that more robust molecular dynamics of remaining D2D3 region in dFlaB would be attributed to the slower mobility. Since monomeric flagellin molecules of bacteria automatically assembles into a filamentous super-structure, it is likely that dFlaB has advantages in terms of pharmaceutical manufacturing and reproducible activities under in vitro and in vivo conditions because of its lower propensity of polymerization.

Though the detailed mode of action for the lowered antigenicity of dFlaB has yet to be investigated, it has been known that B-cell epitopes tend to be less mobile [37, 38] and the low antigenicity of dFlaB may be due to the change in structural flexibility by the 19 amino acids removal. To address this hypothesis, we performed short-period molecular dynamics (MD) simulations. dFlaB was homology-modeled using the same template structures with the same settings as for FlaB (The Tinker package with AMBER99sb and the GB/SA implicit solvent model). Triplicate MD was run per target monomer. Supplementary Fig. 7 shows the root mean square fluctuation (RMSF) of Cα atoms. RMSF values are overall similar, but significant differences were observed at residues right after the truncated region. The flexibility becomes stabilized again at the end of D3. From these observations, we presume that the removal of the 19 amino acids induces high flexibility of D2-D3, which should have hindered antibody recognition [39] (Supplementary Video 1 and Supplementary Data 1).

Given than flagellin is a representative protein-based TLR agonist, the pharmacokinetic property of flagellin might determine the in vivo efficacy of this immunomodulator. In our previous studies, we showed that mucosally (intranasal or intravaginal route) administered FlaB moved to draining lymph nodes (dLNs) within 4–6 h as intact protein aggregates or in association with antigen presenting cells. In the present study, we noted that intranasally administered dFlaB showed equivalent adjuvant activity as WT FlaB in inducing Ag-specific Ab responses, suggesting in vivo stability was not compromised by the deletion of B-cell epitope. It has been reported that biodistribution and antigen delivery to dLN of recombinant protein vaccine is critical for the development of optimal recombinant vaccine [40]. We also observed that in vivo-administered dFlaB similarly distributed in the dLNs as compared with WT FlaB (Fig. 4).

In this study, we developed deimmunized FlaB (dFlaB) by deleting B-cell epitope in the variable region of FlaB. The dFlaB maintains stable TLR5-stimulating activity. We confirmed that repeated immunization of dFlaB does not induce flagellin-specific Abs and dFlaB-adjuvant exert equal efficacy to WT FlaB in inducing influenza vaccine-specific Ab responses. The dFlaB-adjuvanted vaccine confer enhanced protection against lethal influenza challenge. The deimmunizing B cell epitope deletion did not compromise stability and adjuvanticity, while suppressing unwanted antibody responses that may negatively affected vaccine antigen-directed immune responses in repeated vaccinations.

## Methods

### Computational methods

BepiPred 2.0 [20] was used to identify potential B-cell epitopes. Additionally, the Parker hydrophilicity [26] was measured using the IEDB webserver [41]. Structure models of FlaB were built using MODELLER [22]. One hundred models were generated and the best one was selected based on the DOPE potential [23]. The final model was deduced by minimizing with the TINKER molecular dynamics package with AMBER99sb [24] and the GB/SA implicit solvent model [25]. Triplicate molecular dynamics (MD) runs were carried out per each target for one nanosecond. Structural snapshots were taken every 5 picoseconds and thus the RMSF values were calculated from 2,000 structural conformations against the initial minimized structure. FoldX [27] was employed to conduct the in silico alanine scanning on the TINKER minimized model. Side-chains were re-minimized using FoldX before alanine scanning.

### Plasmid construction and recombinant protein production

The expression vector for FlaB^ΔD2D3^ and dFlaB fusion proteins were constructed by crossover-PCR-mediated deletion mutagenesis [42, 43]. Bacterial strains and plasmids used in this study are listed in Table 1. The PCR primers used in this study are listed in Supplementary Table 1. Plasmids were maintained in *Escherichia coli* grown on Luria–Bertani (LB) agar plates with ampicillin (100 μg/ml) or kanamycin (100 μg/ml). To construct the expression vectors for the FlaB^ΔD2D3^ and dFlaB, we designed two sets of primers to amplify upstream or downstream region of *fla*B gene. The primers were synthesized with overhangs recognized by specific restriction enzymes (REs). The upstream and downstream amplicons of *fla*B fragments were ligated by cross-over PCR to produce corresponding nucleotide sequences for FlaB^ΔD2D3^ and dFlaB proteins. The purified PCR products were cloned into pCR2.1 TOPO vector (Invitrogen, Inc., Carlsbad, CA) and fusion fragments were digested with appropriate REs and subcloned into pET30a^+^ plasmids. The DNA sequences of the resulting expression vectors were confirmed by the dideoxy-chain termination sequencing method via the Macrogen Online Sequencing Order System (http://dna.macrogen.com/kor/). The resulting expression plasmids were transformed into competent *E. coli* BL21. Protein expression was induced with 0.1 mM isopropyl-β-D-thiogalactoside (IPTG) for 18 h incubation at 20 °C, and cells were pelleted by centrifugation and stored at −80 °C until use. The bacterial pellets were lysed by 50 mL of lysis buffer (pH 8, 50 mM NaH_2_PO_4_, 300 mM NaCl, and 10 mM imidazole, 0.1% TritonX-100, 0.1% Tween, and 20 µM phenylmethylsulfonyl fluoride). After centrifugation at 35,000 × *g* for 30 min, cell-free supernatant was loaded on a column containing Ni-NTA agarose beads (Qiagen, Hilden, Germany) following manufacturer’s instruction. Wild type recombinant FlaB protein was produced from pCMM250 plasmid which was originally established in our previous study [28] by using pTYB12 expression system (New England Biolabs, Inc., Beverly, MA). Briefly, pCMM250 plasmid was transformed into competent *E. coli* ER2566 and an intein-FlaB fusion protein was induced by 0.5 mM IPTG. To prepare a bacterial lysate for affinity column chromatography, the pellet was resuspended in a lysis buffer (20 mM Tris–Cl pH 7.5, 500 mM NaCl, 1 mM EDTA pH 8.0, 0.1% Triton X-100, 0.1% Tween 20, 20 μM phenylmethylsulfonyl fluoride) and sonicated (Vibra Cell VCX500; Sonics & Materials, Inc., Newtown, CT) on an ice bed. After sonication, recombinant intein-free FlaB was purified by using a chitin column and 50 mM 1,4-dithiothreitol solution in accordance with the manufacturer’s protocol (Chitin Resin, New England Biolabs, Inc.). The purity of the recombinant proteins was confirmed by sodium dodecyl sulfate-polyacrylamide gel electrophoresis (SDS-PAGE), native-PAGE, and subsequent Western blot analysis with an anti-FlaB antibody raised in mice using complete Freund’s adjuvant (Sigma-Aldrich, St. Louis, MO). All blots derived from the same experiment and were processed in parallel. Column-purified protein buffer was exchanged into phosphate buffered saline (PBS) by centrifugal filter tubes (Amicon® Ultra-15 Centrifugal Filter, 10k). Lipopolysaccharide (LPS) contamination was removed by treatment with TritonX-114 (Sigma-Aldrich, St. Louis, MO) and traces of Triton X-114 were removed by treatment with Bio-Beads™ SM-2 (Bio-Rad Laboratories, Inc., Hercules, CA) as per manufacturer’s instructions by incubating with 0.3 g of Bio-beadsTM-2 for 1 ml of protein. The residual LPS content was determined by using the gel-clotting Endosafe LAL kit (Charles River, Charleston, SC). The LPS levels in protein preparations were kept below the Food and Drug Administration (FDA) guideline (less than 0.15 EU/30 g per mouse).

### Determination of TLR5-dependent NF-κB activation

We measured TLR5-dependent NFκB stimulating activity of the recombinant proteins by using HEK-Blue^TM^ hTLR5 cells (InvivoGen, hκb-htlr-5) and HEK-Blue^TM^ Detection (InvivoGen, hb-det2) assay systems following the manufacturer’s instruction. EC_50_ was calculated using triplicate OD 620 nm values for each protein concentration over a wide range of protein concentrations (0.03719 nM to 76.16 nM at the AAT Bioquest website [https://www.aatbio.com/tools/ec50-calculator]).

### Ethics statement

All animal experimental procedures were performed with approval from the Chonnam National University Institutional Animal Care and Use Committee under protocol CNU IACUC-H-2018-66. Animal research facility maintenance and experimental procedures were carried out strictly keeping the guideline of the Animal Welfare Act legislated by Korean Ministry of Agriculture, Food, and Rural Affairs.

### Intranasal immunization

Seven-week-old female BALB/c mice (OrientBio, Seongnam, Korea) were intranasally immunized with vaccine components at 2-week intervals under anesthesia by using Zoletil® 50 (Virbac corporation, Carros, France) and Rompun^TM^ (Bayer AG, Leverkusen, Germany). To evaluate flagellin-specific Ab responses induced by FlaB or dFlaB, mice were immunized with 4 μg FlaB or 4 μg dFlaB seven times at two-week intervals. For the influenza vaccine study, groups of mice were immunized with 10 μl PBS, per each nostril, containing 4 μg dFlaB, 4 μg FlaB, 1.5 μg H3N2 A/Switzerland/9715293/2013 NIB-88 split commercial vaccine (sH3N2; IL-YANG PHARM. Yongin, Korea), 1.5 μg sH3N2 plus 4 μg dFlaB or 1.5 μg sH3N2 plus 4 μg FlaB three times at 2-week intervals. The serum and bronchoalveolar lavage fluid (BALF) were collected from the immunized mice in each group [28] and stored at −80 °C until use.

### In vivo tracing of FlaB or dFlaB

To test in vivo biodistribution of FlaB or dFlaB, BALB/c mice were administered with 100 μg of FlaB-FNR675 or dFlaB-FNR675 through intranasal or footpad route. To prepare fluorescence-conjugated proteins, purified FlaB or dFlaB was mixed with FNR675-NHS ester (BioActs, Korea) at 4 °C and maintained overnight in the dark with stirring. The labeled FlaB-FNR675 or dFlaB-FNR675 was then separated from the unconjugated dye using a centrifugal filter (10 kDa cutoff) (Amicon Ultra®-4, UFC801024), followed by washing (5×) in PBS. Next, the amount of conjugated protein was determined from the calibration curve of the FNR675-NHS ester using a UV-Vis spectrophotometer (UV-2700, Shimadzu, Japan). Six hours after the administration, cervical, inguinal, and popliteal lymph nodes were isolated to assess the biodistribution of the FlaB or dFlaB. The photon signals were collected using a fluorescence labeled organism bioimaging instrument (FOBI; NeoScience company, Korea). and Signal intensities were assessed quantitatively in the specimens by measuring the maximum photons per second per centimeter squared per steradian (p/s/cm2/sr).

### ELISPOT assay of Ab-secreting cells

To evaluate FlaB-specific B cell responses after multiple immunization of the dFlaB or FlaB, we determined IgG- or IgA-secreting plasma cells in bone marrow and memory B cells in spleen [29]. To determine FlaB-specific antibody-secreting cells (ASCs), multi-screen 96-well plates (BD Biosciences) were coated with recombinant FlaB (1 μg/well) overnight at 4 °C. After blocking with RPMI1640 (Thermo Fischer Scientific Inc. Waltham, MA) supplemented with 10% fetal bovine serum (Thermo Fischer Scientific Inc. Waltham, MA), 10^6^ splenocytes or 10^6^ bone marrow cells were added to the FlaB-coated plates, followed by incubation for 24 h for bone marrow cells, or 5 days for spleen cells. The plates were subsequently incubated with HRP-conjugated anti-mouse IgG or IgA according to the manufacture’s protocol, and the spots were visualized with the AEC substrate (BD Biosciences, BD, Franklin Lakes, NJ) and counted with a CTL-Immunospot Analyzer (Cellular Technology, Shaker Heights, OH).

### Determination of sH3N2- or flagellin-specific antibody responses by ELISA and Western blotting analysis

To determine antigen-specific antibody (Ab) titers by ELISA, the serum and BALF were collected from immunized mice. The ELISA plates (Corning Laboratories, Sigma-Aldrich, St. Louis, MO) were coated by incubating with antigens in PBS for 24 h at 4 °C. The plates were washed with sterile distilled water (DW) to remove the unbound-antigen and treated with blocking buffer [0.5% BSA (Sigma-Aldrich, St. Louis, MO), 1 mM EDTA (BIONEER, KOREA) in PBST] at RT for 1 hour. Serially diluted sera or saliva in blocking buffer were added into the plates and incubated for 2 h at RT and then the 5 washes were repeated. The HRP-conjugated anti-mouse IgG or IgA antibodies were used as secondary Abs. The signal was developed with 30 μl of 3,3′5,5′tetramethylbenzidine (TMB) substrate (BD, Franklin Lakes, NJ). The reaction was stopped by the addition of 30 μl of 1 N H_2_SO_4_. The optical density was measured with a microplate reader (Molecular Devices Corp., Menlo Park, CA) at 450 nm. The titers were expressed as the reciprocal log2 value of the dilution that yielded 2-fold higher values of optical density at 450 nm than the no serum blank well.

For Western blotting, the recombinant proteins were separated by SDS-PAGE or native-PAGE and transferred onto nitrocellulose membranes (Amersham, Merck KGaA, Darmstadt, Germany). Anti-sera or BALF were diluted in PBS with 0.05% Tween-20 were incubated with the respective membranes for 2 h at room temperature (RT) to probe corresponding proteins. The horseradish peroxidase (HRP)-conjugated secondary antibody (Dako, Agilent Technologies, Inc., Santa Clara, CA) was used to visualize proteins following the manufacture’s instruction.

### Virus plaque reduction neutralization test (PRNT)

Anti-sera were incubated with receptor destroying enzyme (Cosmos Biomedical Ltd, Derbyshire, United Kingdom) for 24 h at 37 °C and then heat inactivated at 56 °C for 1 hour. Virus neutralizing activity was determined [44–47]. Briefly, the two-fold serially diluted heat inactivated serum samples were incubated with equal volume of influenza virus suspension (30–40 PFU) at 37 °C with 5% CO_2_ atmosphere for 2 h. The mixture of serum and virus were adsorbed to 12‐well plates containing confluent Madin‐Darby canine kidney cells. The agar‐overlaid plates were incubated at 37 °C in a 5% CO_2_ incubator. Four days after the virus infection, to visualize plaques, the plates were stained with 0.03% crystal violet.

### Lethal challenge with live influenza virus

To test whether dFlaB- or FlaB-adjuvanted influenza vaccines elicit protective immune responses in a viral infection model, we carried out challenge experiments. Groups of mice (*n* = 9–10) were vaccinated with 4 μg dFlaB plus 0.2 or 0.08 μg H1N1 A/Brisbane/59/07 split vaccine (Green Cross, Hwasun, Korea) (sH1N1 + dFlaB) or 4 μg FlaB plus 0.2 or 0.08 μg sH1N1 (sH1N1 + FlaB). Two weeks after the 3rd immunization, the vaccinated mice were infected with live 2.4x LD_50_ of A/Brisbane/59/07 homologous influenza virus strain under anesthetic condition. After the lethal viral infection, the challenged mice were monitored daily for 2 weeks [11]. Mice that lost 30% of their total weight were euthanized. The A/Brisbane/59/07 (H1N1) viruses were amplified by infecting Madin Darby Canine (MDCK) cells (American Type Culture Collection, Manassas, VA, USA) [48, 49]. Briefly, MDCK cells were maintained in Minimum Essential Media/Earle’s Balanced Salt Solution (MEM/EBSS) (HyClone Laboratories, Marlborough, USA) containing 10% fetal bovine serum (Gibco), penicillin (100 U/ml), and streptomycin (100 μg/ml) at 37 °C in 5% CO_2_ atmosphere. To propagate A/Brisbane/59/07 virus, the MDCK cells were infected with the virus in serum-free MEM/EBSS for 2 h at RT. Then the cells were incubated with MEM/EBSS containing 5% Bovine Serum Albumin (MP biomedicals 160069, Irvine, CA) and 1 μg/ml 1-tosylamide-2-phenylethyl chloromethyl ketone (TPCK)-trypsin (Sigma Aldrich, St. Louis, MO) media up to 2 days. When cytophatic effect was evident, the viruses were harvested by centrifugation.

### Statistical analyses

The results are expressed as the mean ± standard error of the mean (SEM) unless otherwise stated. Mann-Whitney or unpaired t-test was used to compare two groups. Statistical significance for survival and body weight changes were calculated by Log-rank (Mantel-Cox) test and two-way ANOVA, respectively. Statistical analyses were performed using the Prism 8.00 software for Windows (GraphPad software, San Diego, CA). *P* values < 0.05 was considered statistically significant.

### Reporting summary

Further information on research design is available in the Nature Research Reporting Summary linked to this article.

## Supplementary information

Reporting Summary

Supplementary Information

Supplementary Movie 1

Supplementary Data 1

## Data Availability

All data generated or analysed during this study are included in this published article and its Supplementary Information files.
