# Supplementary material for: Deimmunization of flagellin for repeated administration as a vaccine adjuvant
Source: NPJ Vaccines. 2021 Sep 13;6:116. doi: 10.1038/s41541-021-00379-4 (PMC8438039; doi:10.1038/s41541-021-00379-4)
Supplement: Supplementary file 1 — Reporting Summary [file 41541_2021_379_MOESM1_ESM.pdf]

nature portfolio

Corresponding author(s): Joon Haeng Rhee and Shee Eun Lee

Last updated by author(s): Jul 28, 2021

Reporting Summary

Nature Portfolio wishes to improve the reproducibility of the work that we publish. This form provides structure for consistency and transparency in reporting. For further information on Nature Portfolio policies, see our Editorial Policies and the Editorial Policy Checklist.

Statistics

For all statistical analyses, confirm that the following items are present in the figure legend, table legend, main text, or Methods section.

n/a	Confirmed	
☐	☒	The exact sample size (*n*) for each experimental group/condition, given as a discrete number and unit of measurement
☐	☒	A statement on whether measurements were taken from distinct samples or whether the same sample was measured repeatedly
☐	☒	The statistical test(s) used AND whether they are one- or two-sided<br>*Only common tests should be described solely by name; describe more complex techniques in the Methods section.*
☐	☒	A description of all covariates tested
☐	☒	A description of any assumptions or corrections, such as tests of normality and adjustment for multiple comparisons
☐	☒	A full description of the statistical parameters including central tendency (e.g. means) or other basic estimates (e.g. regression coefficient) AND variation (e.g. standard deviation) or associated estimates of uncertainty (e.g. confidence intervals)
☐	☒	For null hypothesis testing, the test statistic (e.g. *F*, *t*, *r*) with confidence intervals, effect sizes, degrees of freedom and *P* value noted<br>*Give P values as exact values whenever suitable.*
☒	☐	For Bayesian analysis, information on the choice of priors and Markov chain Monte Carlo settings
☒	☐	For hierarchical and complex designs, identification of the appropriate level for tests and full reporting of outcomes
☒	☐	Estimates of effect sizes (e.g. Cohen's *d*, Pearson's *r*), indicating how they were calculated

Our web collection on statistics for biologists contains articles on many of the points above.

Software and code

Policy information about availability of computer code

Data collection	1) Molecular dynalics: Tinker molecular dynamic package (ver 8.7)<br>5) Statistical analysis: Graphpad Prism 8<br>6) Western blot analysis: iBrightTM CL1000, Firmware Version: 1.2.5<br>7) Fluorescence images analysis: NEOimage FOBI, Version 3.3<br>8) Determine NF-kB activation: Spectrophotometer (SpectraMax 190, Molecular Devices Corp., Menlo Park, CA)<br>9) ELISPOT analysis: ImmunoSpot®, Version 6.4.87, Cellular Technology, Shaker Heights, OH.<br>10) ELISA analysis: Spectrophotometer (SpectraMax 190, Molecular Devices Corp., Menlo Park, CA)
Data analysis	PyMol (2.4) was employed to visualize and generate the molecular dynamics videos. This paper, all statistical analysis were calculated by using the Graphpad Prism 8. iBrightTM CL1000, Firmware Version: 1.2.5 was used for western blot analysis. The fluorescence image analysis was performed by using NEOimage FOBI, Version 3.3. the Determination of NF-kB activation activity was read by Spectrophotometer (SpectraMax 190, Molecular Devices Corp., Menlo Park, CA) HEK-Blue detection with OD620. The ImmunoSpot®, Version 6.4.87, Cellular Technology, Shaker Heights, OH was used for ELISPOT analysis. The ELISA analysis was read by using Spectrophotometer (SpectraMax 190, Molecular Devices Corp., Menlo Park, CA).

For manuscripts utilizing custom algorithms or software that are central to the research but not yet described in published literature, software must be made available to editors and reviewers. We strongly encourage code deposition in a community repository (e.g. GitHub). See the Nature Portfolio guidelines for submitting code & software for further information.

Data

Policy information about availability of data

All manuscripts must include a data availability statement. This statement should provide the following information, where applicable:

- Accession codes, unique identifiers, or web links for publicly available datasets
- A description of any restrictions on data availability
- For clinical datasets or third party data, please ensure that the statement adheres to our policy

Provide your data availability statement here.

Field-specific reporting

Please select the one below that is the best fit for your research. If you are not sure, read the appropriate sections before making your selection.

☒ Life sciences ☐ Behavioural & social sciences ☐ Ecological, evolutionary & environmental sciences

For a reference copy of the document with all sections, see nature.com/documents/nr-reporting-summary-flat.pdf

Life sciences study design

All studies must disclose on these points even when the disclosure is negative.

Sample size	We performed preliminary experiments to determined sufficient sample size for each experiment set.
Data exclusions	No samples or animal were excluded from analyses.
Replication	All experiment finding were performed at least 2 times reproducibly. The number of samples for each experiment indicated in figure legend. The data shown in the figure panels are the mean of all independent repeated experiments. SDS-PAGE, western blot picture, fluorescence image are from a representative experiment. all repeated experiments were successful.
Randomization	For in vitro experiment, cells culture were chosen for different treatment randomly and all experiments were performed at least 2 times. For animal experiments, Seven-week-old female Balb/c mice were selected for intranasal immunization and virus challenge.
Blinding	ELISPOT assay was performed by individuals (Koemchhoy Khim, Sao Puth) who were blinded to nature of mice under analysis.

Reporting for specific materials, systems and methods

We require information from authors about some types of materials, experimental systems and methods used in many studies. Here, indicate whether each material, system or method listed is relevant to your study. If you are not sure if a list item applies to your research, read the appropriate section before selecting a response.

Materials & experimental systems

n/a	Involved in the study	
☐	☒	Antibodies
☐	☒	Eukaryotic cell lines
☒	☐	Palaeontology and archaeology
☐	☒	Animals and other organisms
☒	☐	Human research participants
☒	☐	Clinical data
☒	☐	Dual use research of concern

Methods

n/a	Involved in the study	
☒	☐	ChIP-seq
☒	☐	Flow cytometry
☒	☐	MRI-based neuroimaging

Antibodies

Antibodies used

Western blotting assay
Polyclonal Rabbit Anti-Mouse Immunoglobulins/HRP (1:2000; Dako Denmark A/S; P0260; Lot#20066043)
Goat Anti-Mouse IgG(H+L)-HRP (1:1000; SouthernBiotech; 1036-05; Lot#D4913-XC08D)

ELISA assay
Goat Anti-Mouse IgA-HRP (1:1000; SouthernBiotech; 1040-05; Lot#J4416-M729)
Goat Anti-Mouse IgG(H+L)-HRP (1:2000; SouthernBiotech; 1036-05; Lot#D4913-XC08D)
Goat Anti-Mouse IgG1-HRP (1:2000; SouthernBiotech; 1071-05; Lot#B5312-W089)
Goat Anti-Mouse IgG2a-HRP (1:2000; SouthernBiotech; 1081-05; Lot#J0216-VH671)

Validation	All antibodies used in this study were validated by the suppliers as follows:<br>Polyclonal Rabbit Anti-Mouse Immunoglobulins/HRP (1:2000; Dako Denmark A/S; P0260; Lot#20066043) for WB: species (Mouse), application (ELISA), manufacturer's website (https://www.agilent.com/search/?Ntt=p0260)<br>Goat Anti-Mouse IgG(H+L)-HRP (1:1000; SouthernBiotech; 1036-05; Lot#D4913-XC08D) for WB: species (Mouse), application (ELISA), manufacturer's website (https://www.southernbiotech.com/?catno=1036-05&type=Polyclonal#&panel2-1)<br>Goat Anti-Mouse IgA-HRP (1:1000; SouthernBiotech; 1040-05; Lot#J4416-M729) for ELISA: species (Mouse), application (WB), manufacturer's website (https://www.southernbiotech.com/?catno=1040-05&type=Polyclonal#&panel1-1&panel2-1)<br>Goat Anti-Mouse IgG(H+L)-HRP (1:1000; SouthernBiotech; 1036-05; Lot#D4913-XC08D) for ELISA: species (Mouse), application (WB), manufacturer's website (https://www.southernbiotech.com/?catno=1036-05&type=Polyclonal#&panel2-1)<br>Goat Anti-Mouse IgG1-HRP (1:2000; SouthernBiotech; 1071-05; Lot#B5312-W089) for ELISA: species (Mouse), application (WB), manufacturer's website (https://www.southernbiotech.com/?catno=1071-05&type=Polyclonal#&panel2-1)<br>Goat Anti-Mouse IgG2a-HRP (1:2000; SouthernBiotech; 1081-05; Lot#J0216-VH671) for ELISA: species (Mouse), application (WB), manufacturer's website (https://www.southernbiotech.com/?catno=1081-05&type=Polyclonal#&panel2-1)

Eukaryotic cell lines

Policy information about cell lines

Cell line source(s)	HEK-BlueTM hTLR5 cells from InvivoGen (Cat No. hκb-htlr-5)
Authentication	This cell line was not Authenticated by us.
Mycoplasma contamination	We confirmed that cell line used was negative for mycoplasma contamination.
Commonly misidentified lines<br>(See ICLAC register)	No commonly misidentified cell lines were used.

Animals and other organisms

Policy information about studies involving animals; ARRIVE guidelines recommended for reporting animal research

Laboratory animals	Seven-week old Balb/c female mice (BALB/cAnNCrlOri) were obtained from the Orient Bio (Orient Bio Co., Republic of Korea).
Wild animals	This study did not involve wild animals.
Field-collected samples	This study did not involve animals collected from the field.
Ethics oversight	All animal experimental procedures were performed with approval from the Chonnam National University Institutional Animal Care and Use Committee under protocol CNU IACUC-H-2018-66. Animal research facility maintenance and experimental procedures were carried out strictly keeping the guideline of the Animal Welfare Act legislated by Korean Ministry of Agriculture, Food and Rural Affairs.

Note that full information on the approval of the study protocol must also be provided in the manuscript.
