# Supplementary material for: Deimmunization of flagellin for repeated administration as a vaccine adjuvant
Source: NPJ Vaccines. 2021 Sep 13;6:116. doi: 10.1038/s41541-021-00379-4 (PMC8438039; doi:10.1038/s41541-021-00379-4)

Methods for supplementary Figure 4.

Determination of long-term stability of FlaB, dFlaB and FlaB$^{\Delta D2D3}$ **by measuring TLR5-dependent NF-κB activation.**

293T cells were seeded in 24-well plates at 2 x 10^5/well and incubated for 24h. Subsequently, cells were transfected with p3x Flag-hTLR5 (100 ng/well), the pNFκB-luc (100 ng/well), and 50 ng/well of pCMV-β-Gal using 5 μl/well of Effectene (Qiagen, Hilden Germany). Twenty-four hours after transfection, the culture media were removed and replaced by serum-free Gibco® DMEM (Thermo Fischer Scientific Inc. Waltham, MA). The cells were treated with FlaB, dFlaB, or FlaB$^{\Delta D2D3}$ and incubated for 24 h. The PBS-treated cells were used as the control group. The medium was removed and cells were treated with 1x lysis buffer 100 μl/well (Promega, Madison, WI) and kept at room temperature for 1h. To determine NF-κB-activation, 30 μl of cell lysate was transferred to 96-well opaque plate for the measurement of luciferase activity while another 30 μl to of cell lysate was transferred to 96-well cell culture plate for β-Gal activity measurement. Luciferase activity was normalized to LacZ expression using the control expression plasmid pCMV-β-Gal (Clontech, Takara Bio, Kyoto, Japan). The luciferase activity was measured by a luminometer (MicroLumatPlus LB 96V; Berthold, Wilbad, Germany) while β-Gal activity was read by a microplate reader (Molecular Devices Corp., Menlo Park, CA) at 420 nm.

Supplementary information

Supplementary Figure 1. Monomeric (left) and oligomeric (right) structures of FlaB variants. **(A)** WT FlaB, **(B)** dFlaB and **(C)** FlaB$^{\Delta D2D3}$. ND2-D3-CD2 domains (D2D3) are colored in red and the removed 19 residues are in blue. D2D3 are solvent exposed in both monomeric and oligomeric forms.

Supplementary Figure 2. Development of recombinant dFlaB and FlaB$^{\Delta D2D3}$. Recombinant plasmids were constructed as described in Materials and Methods. The vector maps and amino acid sequences of recombinant proteins are shown

Supplementary Figure 3. MALDI-TOF analysis report. Called up peptide sequences by MALDI-TOP analyses are shown in bold red.

Supplementary Figure 4. Determination of TLR5-dependent NF-κB stimulating activity by FlaB, dFlaB and FlaB$^{\Delta D2D3}$. The relative luciferase activities in the cell extracts were analyzed by a dual-luciferase reporter assay system and normalized using the pCMV-β-Gal plasmid as a control. The same molar ratio of proteins was used, and PBS was used as a negative control.

Supplementary Figure 5. sH3N2-specific antibody response after intranasal vaccination. Groups of mice were intranasally vaccinated with PBS (PBS), 4 μg FlaB (FLaB), 4 μg dFlaB (dFlaB), 1.5 μg H3N2 A/Switzerland/9715293/2013 NIB-88 split vaccine (sH3N2), 1.5 μg sH3N2 plus 4 μg FlaB (sH3N2+FlaB), or 1.5 μg sH3N2 plus 4 μg dFlaB (sH3N2+dFlaB) three times at two-week intervals. Two weeks after the last immunization, serum was collected and sH3N2-specific serum IgG_1 or IgG_{2a} titers were determined by ELISA.

Supplementary Figure 6. sH1N1-specific antibody response after intranasal vaccination. Groups of mice were intranasally immunized with PBS, FlaB, dFlaB, H1N1 A/Brisbane/59/07 split vaccine (sH1N1), sH1N1 plus FlaB (sH1N1+FlaB), or sH1N1 plus dFlaB (sH1N1+dFlaB) three times at two-week intervals. Two weeks after the last immunization, serum was collected and sH1N1-specific serum IgG titer weas determined by ELISA.

Supplementary Figure 7. RMSF plots for FlaB and dFlaB. Three molecular dynamics simulations were performed for each variant. Though overall RMSF patterns are similar each other,

notable changes are observed in the truncated region (shaded in blue). The high fluctuation due to the removal of 19 amino acids may have resulted in low antigenicity of dFlaB.

Supplementary Video 1. Representative molecular dynamics simulations of (A) WT FlaB and **(B)** dFlaB. ND2-D3-CD2 domains (D2D3) are colored in red and the removed 19 residues are in blue.

Supplementary Data 1. BepiPred-2.0, FoldX alanine scanning and the Parker hydrophobicity scales of FlaB.

Supplementary Table 1. PCR primer used in this study

Gene	Direction	Sequence
dFlaB	Up/Forward	5’ GGAATTC **CATATG** ATGGCAGTGAATGTAAATAC 3’
	Up/Reverse	5’ TGCAATCGTCAAGTC CACGCCGCCCATCAT 3’
	Down/Forward	5’ ATGATGGGCGGCGTG GACTTGACGATTGCA 3’
	Down/Reverse	5’ CGG **CTCGAG** GCCTAGTAGACTTAGCGCTG 3’
$FlaB^{\Delta D2D3}$	Up/Forward	5’ GGAATTC **CATATG** ATGGCAGTGAATGTAAATAC 3’
	Up/Reverse	5’ CTCTTGCGCACCTTG CACGTTGTCAGAGCG 3’
	Down/Forward	5’ CGCTCTGACAACGTG CAAGGTGCGCAAGAG 3’
	Down/Reverse	5’ CGG **CTCGAG** GCCTAGTAGACTTAGCGCTG 3’

Underlined sequences indicate the restriction enzyme recognition sites.

Supplementary Figure 1

a

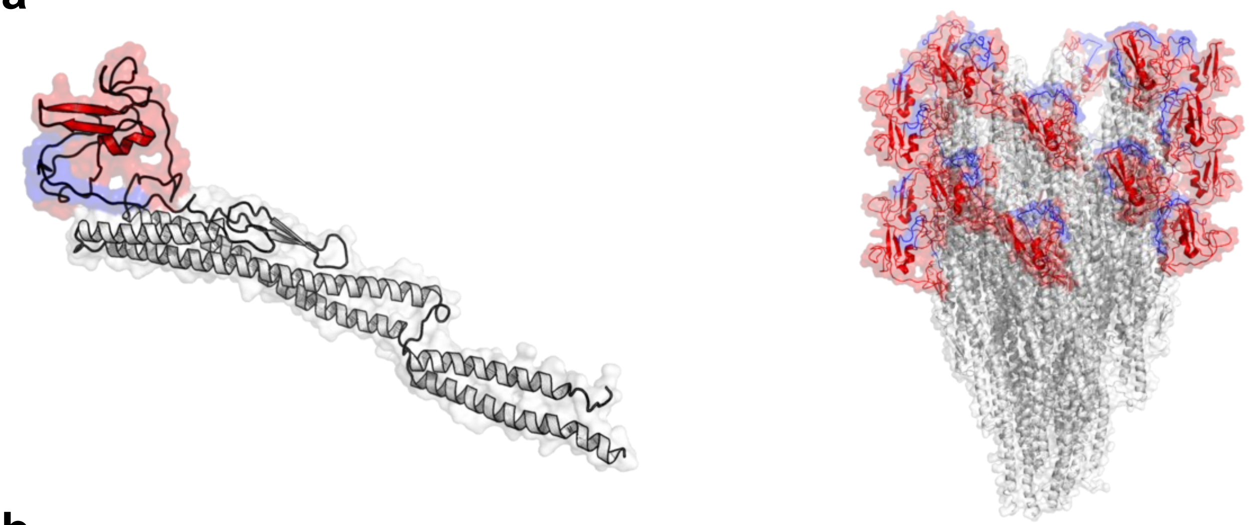

b

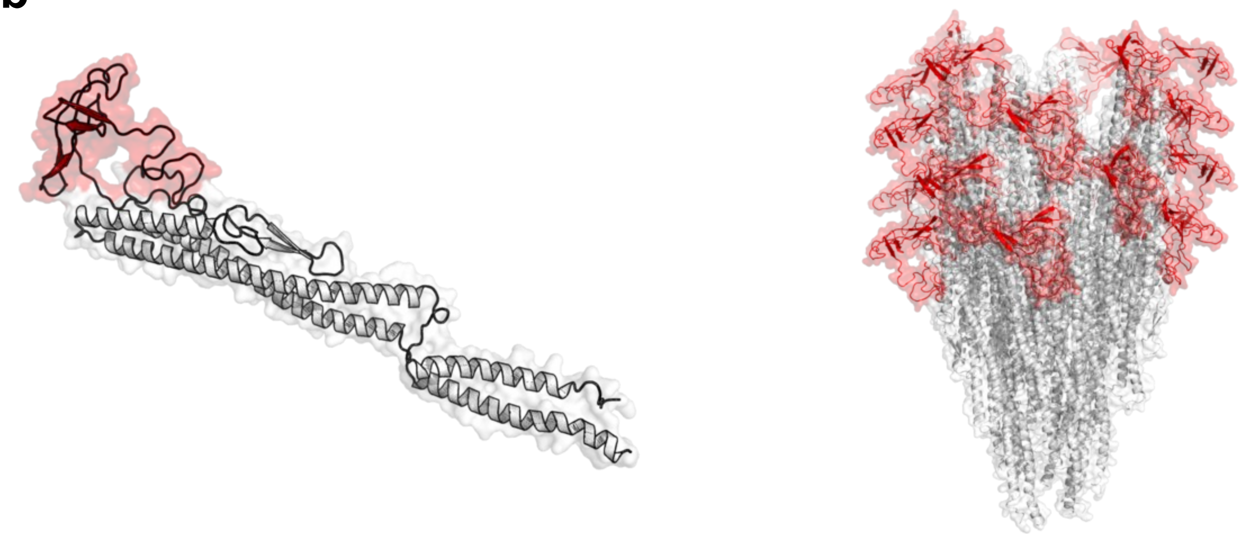

c

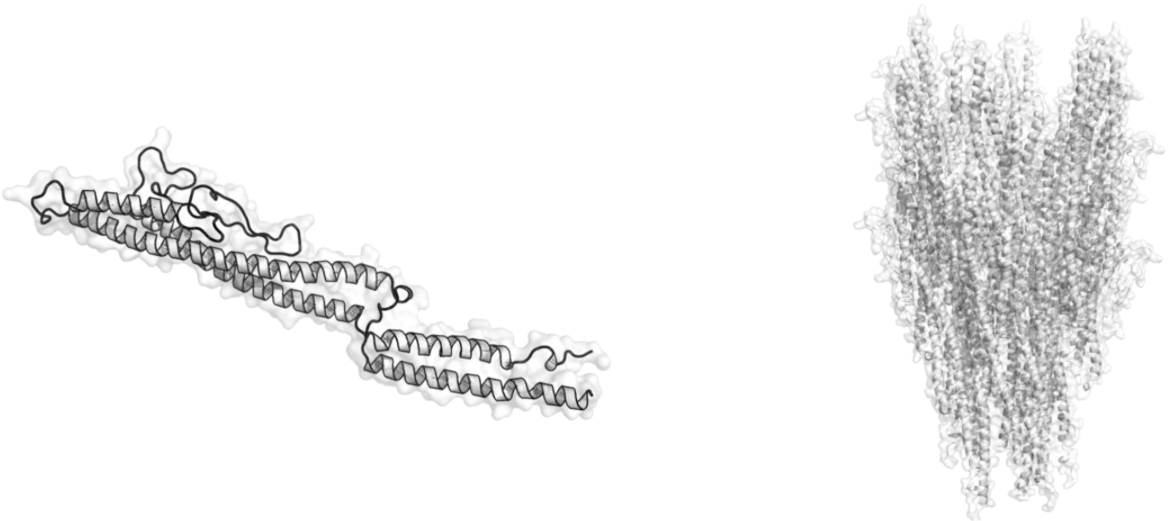

Supplementary Figure 2

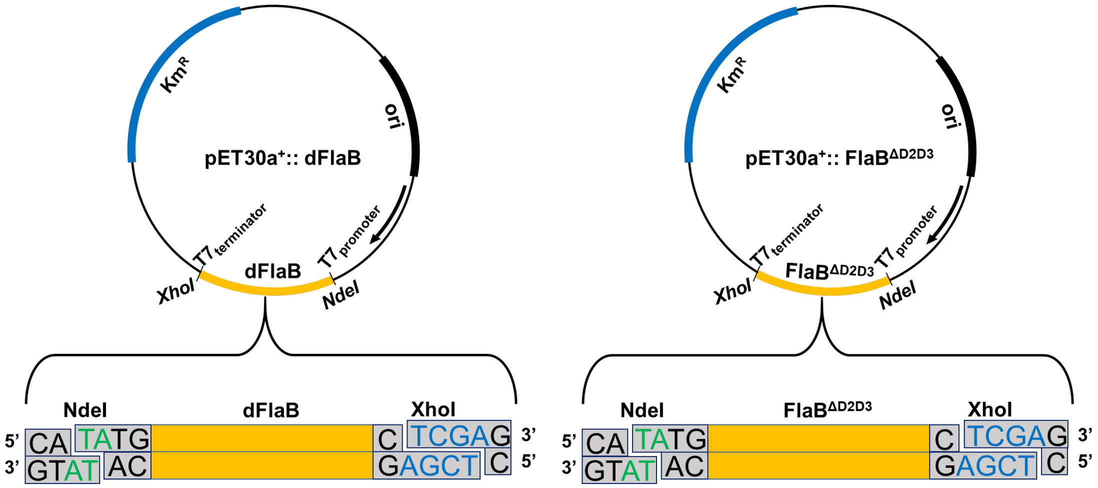

dFlaB

HMMAVNVNTNVAAMTAQRYLNNANSAQQTSMERLSSGFKINSAKDDAAGLQISNRLNVQSRGLDVAVR
NANDGISIAQTAEGAMNETTNILQRMRDLSLQSANGSNSKSERVAIQEEVTALNDELNRIAETTSFGGNK
LLNGTYGTKAMQIGADNGEAVMLSLKDMRSDNVMMGGVSYQAEEGKDKNWNVAAGDNDLTIALTDSF
GNEQEIEINAKAGDDIEELATYINGQTDLVKASVGEGGKLQIFAGNNKVQGEIAFSGSLAGELGLGEGKN
VTVDTIDVTTVQGAQESVAIVDAALKYVDSHRAELGAFQNRFNHAISNLDNINENVNASKSRIKDTDFAK
ETTQLTKTQILSQASSSILAQAKQAPNSALSLLGLEHHHHHH

FlaB$^{\Delta D2D3}$

HMMAVNVNTNVAAMTAQRYLNNANSAQQTSMERLSSGFKINSAKDDAAGLQISNRLNVQSRGLDVAVR
NANDGISIAQTAEGAMNETTNILQRMRDLSLQSANGSNSKSERVAIQEEVTALNDELNRIAETTSFGGNK
LLNGTYGTKAMQIGADNGEAVMLSLKDMRSDNVMMGGVSYQAEEGKDKNWNVAAGDNDLTIALTDSF
GNEQEIEINAKAGDDIEELATYINGQTDLVKASVGEGGKLQIFAGNNKVQGEIAFSGSLAGELGLGEGKN
VTVDTIDVTTVQGAQESVAIVDAALKYVDSHRAELGAFQNRFNHAISNLDNINENVNASKSRIKDTDFAK
ETTQLTKTQILSQASSSILAQAKQAPNSALSLLGLEHHHHHH

***Underlined Red: deleted amino acid**

Supplementary Figure 3

Protein: FlaB

Enzyme: Trypsin: cuts C-term side of KR unless next residue is P.
Fixed modifications: Carbamidomethyl (C)
Variable modifications: Oxidation (M)

Protein sequence coverage: 70%

Matched peptides shown in ***bold red***.

1 MAVNVNTNVA AMTAQR**YLNN** **ANSAQQTSME** RLSSGFK**INS** **AKDDAAGLQI**
51 **SNR**LNVQSRG LDVAVR**NAND** **GISIAQTAEG** **AMNETTNILQ** **R**MRDLSLQSA
101 NGSNSKSER**V** **AIQEEVTALN** **DELNRIAETT** **SFGGNK**LLNG TYGTK**AMQIG**
151 **ADNGEAVMLS** **LKDMRSDNVM** **MGGVSYQAEE** **GKDKNWNVAA** **GDNDLTIALT**
201 **DSFGNEQEIE** **INAKAGDDIE** **ELATYINGQT** **DLVK**ASVGEG GK**LQIFAGNN**
251 **KVQGEIAFSG** **SLAGELGLGE** **GKNVTVDTID** **VTTVQGAQES** **VAIVDAALK**Y
301 VDSHR**AELGA** **FQNRFNHAIS** **NLDNINENVN** **ASK**SR**IKDTD** **FAK**ETTQLTK
351 TQILSQASSS ILAQAKQAPN SALSLLG

Unformatted sequence string: 377 residues (for pasting into other applications).

Protein: dFlaB

Enzyme: Trypsin: cuts C-term side of KR unless next residue is P.
Fixed modifications: Carbamidomethyl (C)
Variable modifications: Oxidation (M)

Protein sequence coverage: 61%

Matched peptides shown in ***bold red***.

1 MAVNVNTNVA AMTAQR**YLNN** **ANSAQQTSME** RLSSGFK**INS** **AKDDAAGLQI**
51 **SNR**LNVQSRG LDVAVR**NAND** **GISIAQTAEG** **AMNETTNILQ** **R**MRDLSLQSA
101 NGSNSKSER**V** **AIQEEVTALN** **DELNRIAETT** **SFGGNK**LLNG TYGTK**AMQIG**
151 **ADNGEAVMLS** **LKDMR**SDNVM MGGVSYQAEE GKDKNWNVAA GDNDLTIALT
201 DSFGNEQEIE INAK**AGDDIE** **ELATYINGQT** **DLVK**ASVGEG GK**LQIFAGNN**
251 **KVQGEIAFSG** **SLAGELGLGE** **GKNVTVDTID** **VTTVQGAQES** **VAIVDAALK**Y
301 VDSHR**AELGA** **FQNRFNHAIS** **NLDNINENVN** **ASK**SR**IKDTD** **FAK**ETTQLTK
351 **TQILSQASSS** **ILAQAK**QAPN SALSLLG

Unformatted sequence string: 377 residues (for pasting into other applications).

Protein: FlaBΔD2D3

Enzyme: Trypsin: cuts C-term side of KR unless next residue is P.
Fixed modifications: Carbamidomethyl (C)
Variable modifications: Oxidation (M)

Protein sequence coverage: 40%

Matched peptides shown in ***bold red***.

1 MAVNVNTNVA AMTAQR**YLNN** **ANSAQQTSME** RLSSGFK**INS** **AKDDAAGLQI**
51 **SNR**LNVQSRG LDVAVR**NAND** **GISIAQTAEG** **AMNETTNILQ** **R**MRDLSLQSA
101 NGSNSKSER**V** **AIQEEVTALN** **DELNRIAETT** **SFGGNK**LLNG TYGTK**AMQIG**
151 **ADNGEAVMLS** **LK**DMRSDNVM MGGVSYQAEE GKDKNWNVAA GDNDLTIALT
201 DSFGNEQEIE INAKAGDDIE ELATYINGQT DLVKASVGEG GKLQIFAGNN
251 KVQGEIAFSG SLAGELGLGE GKNVTVDTID VTTVQGAQES VAIVDAALKY
301 VDSHR**AELGA** **FQNRFNHAIS** **NLDNINENVN** **ASK**SR**IKDTD** **FAK**ETTQLTK
351 **TQILSQASSS** **ILAQAK**QAPN SALSLLG

Unformatted sequence string: 377 residues (for pasting into other applications).

Supplementary Figure 4

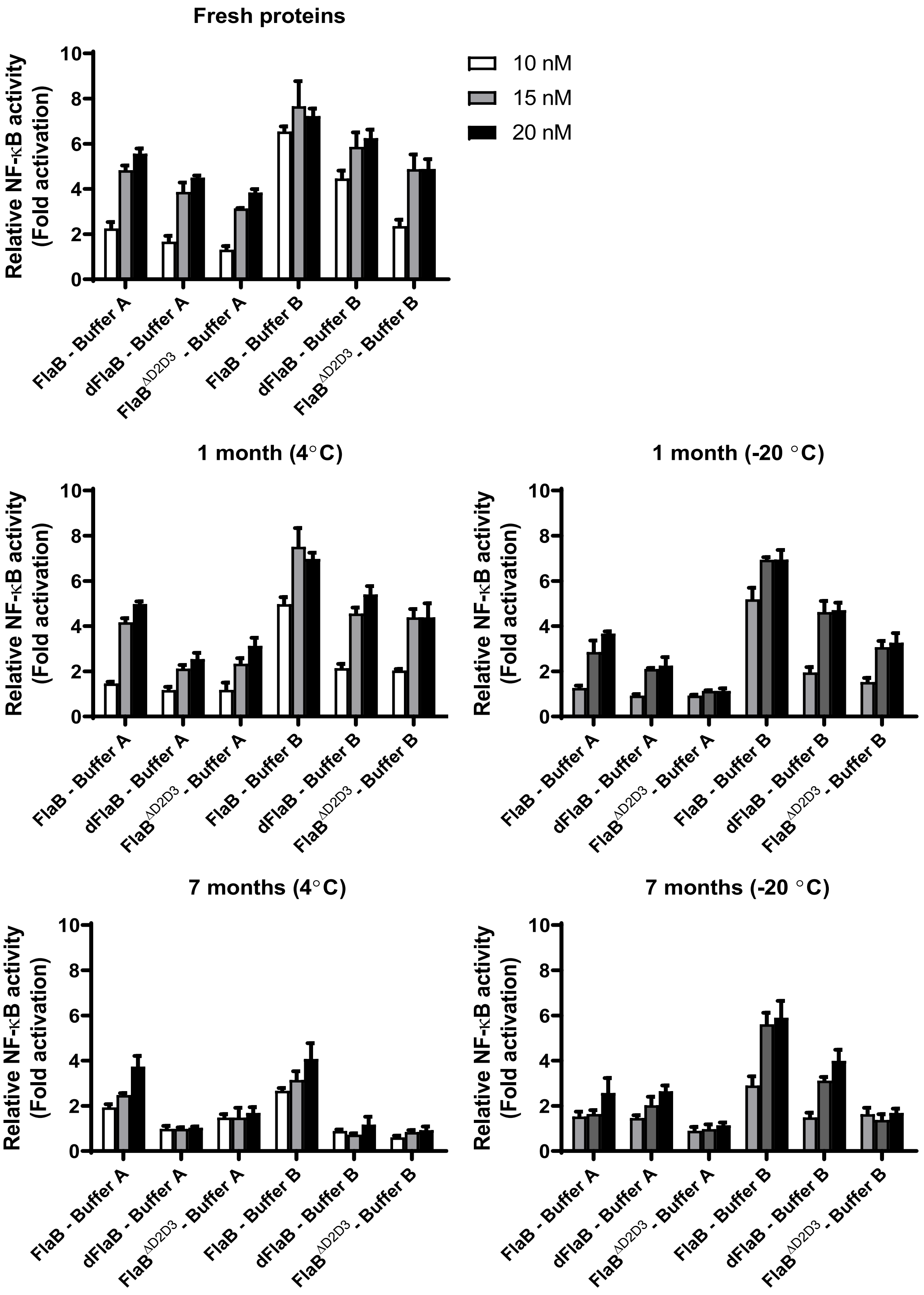

Supplementary Figure 5

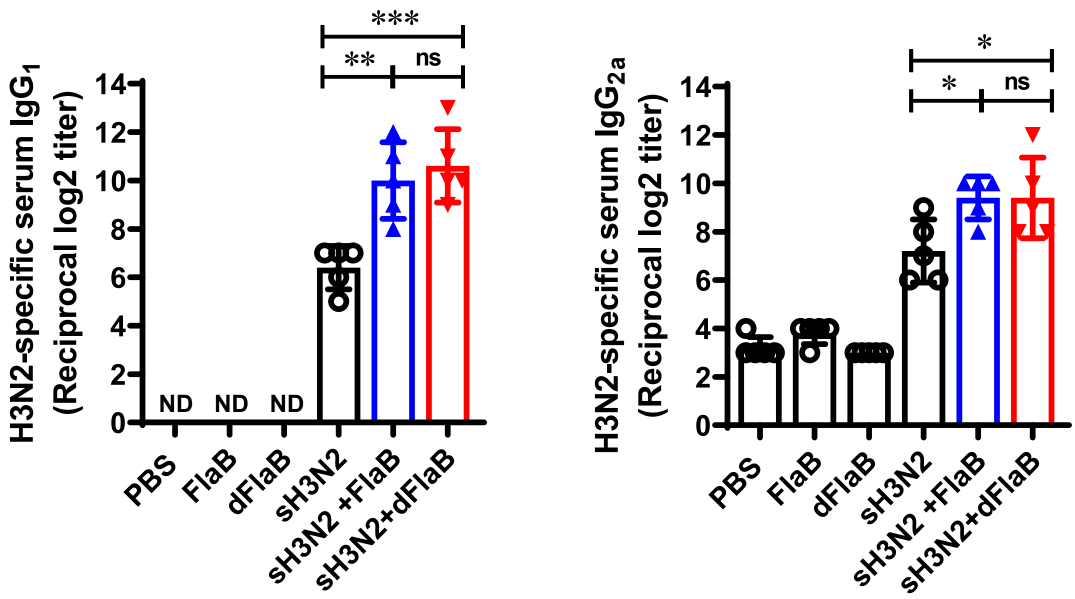

Supplementary Figure 6

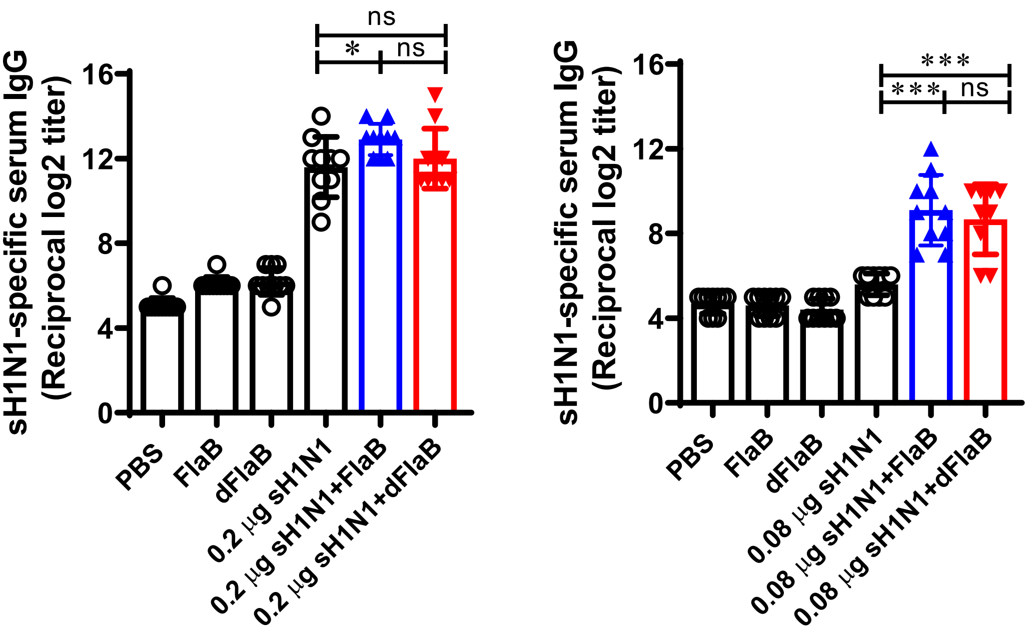

Supplementary Figure 7

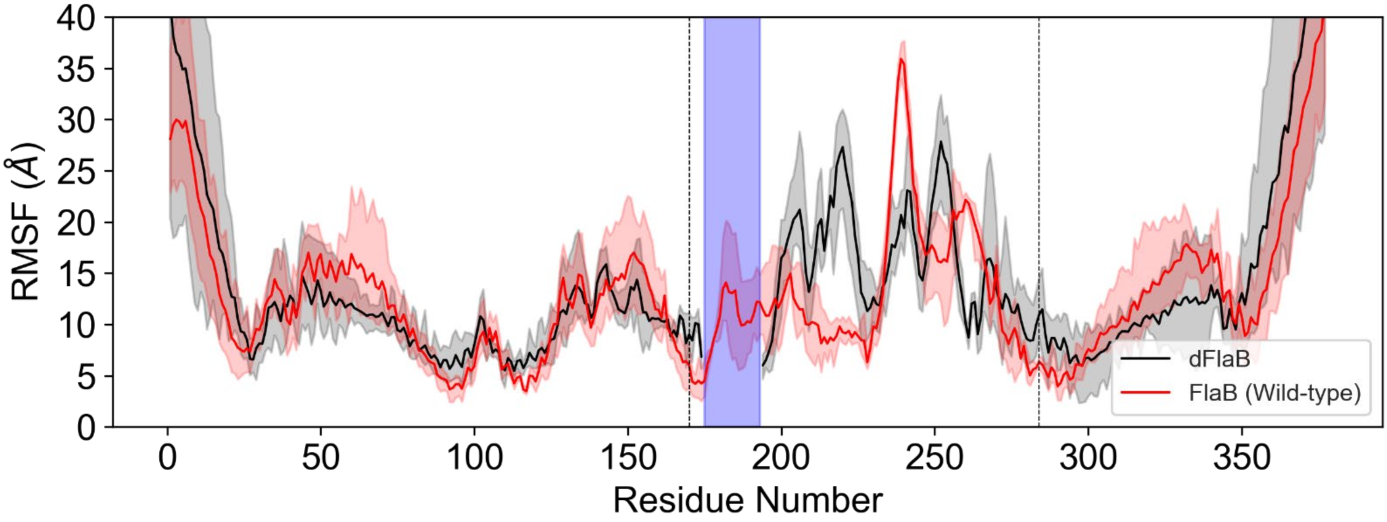

Figure 2a: Unprocessed W blot

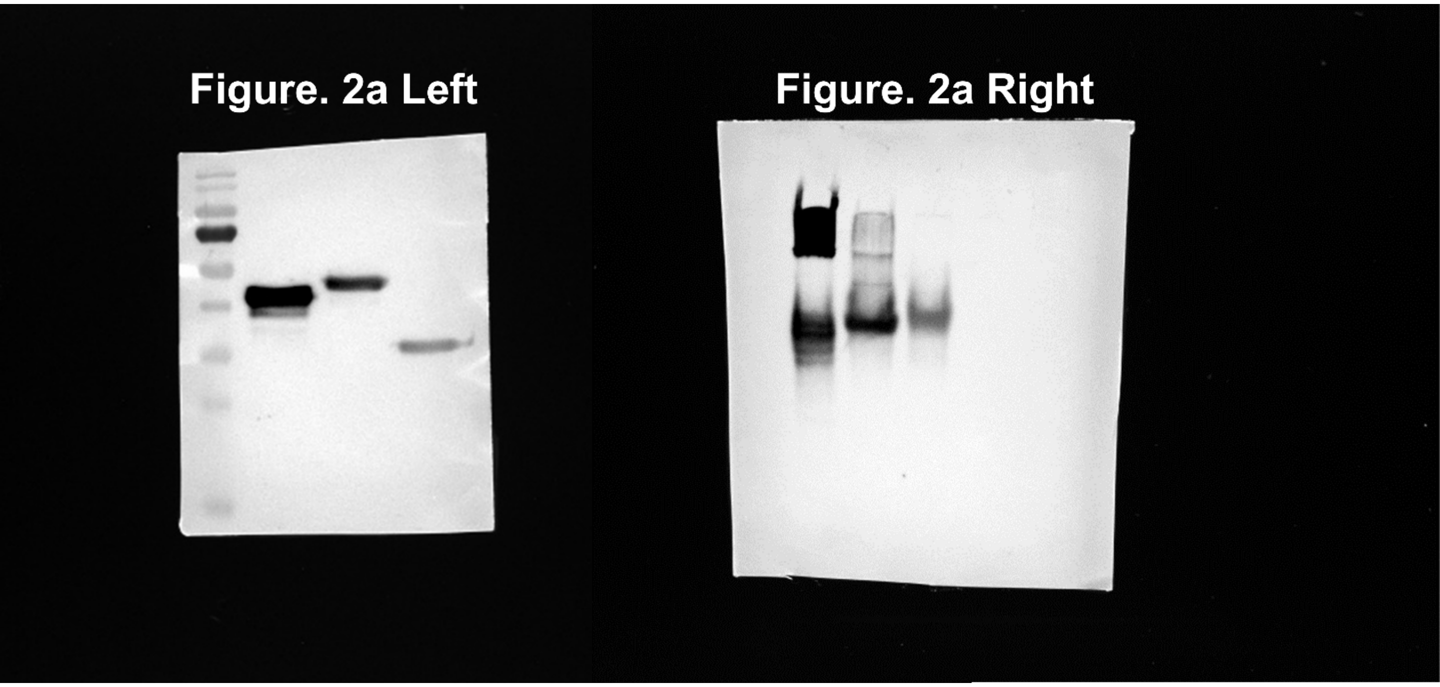

Figure 3c: Unprocessed W blot

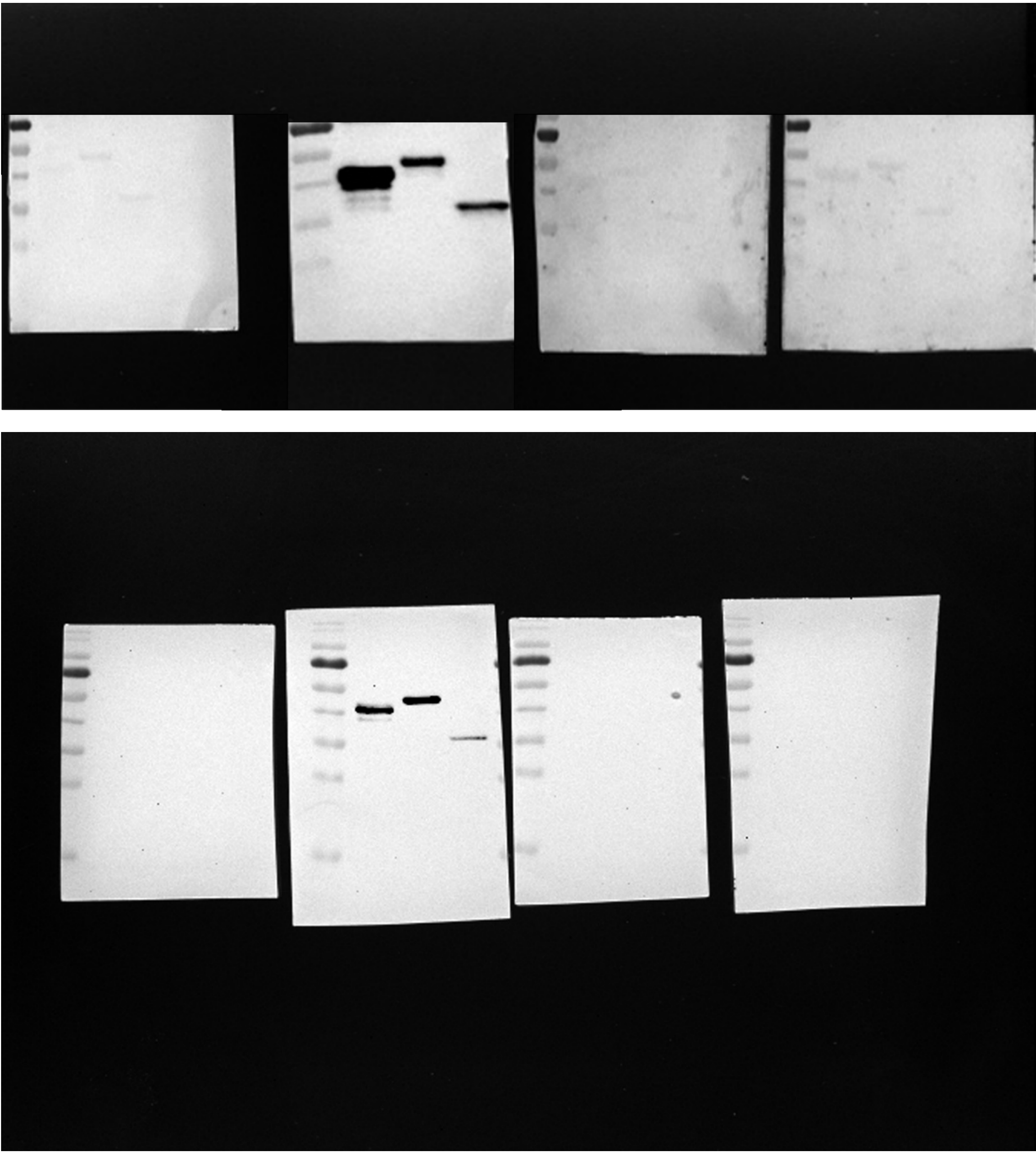

Figure 5b: Unprocessed W blot

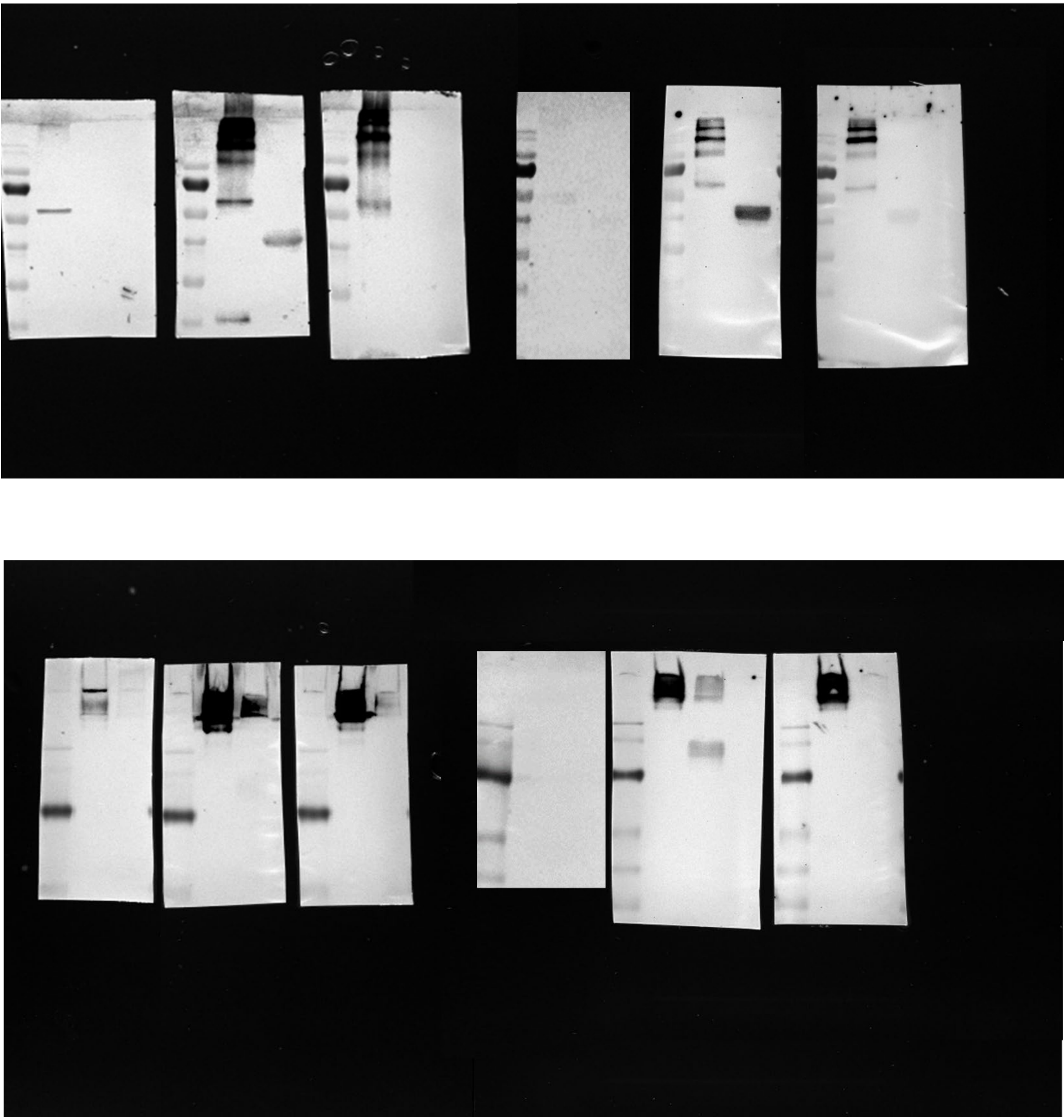

Supplement: Supplementary file 2 — Supplementary Information [file 41541_2021_379_MOESM2_ESM.pdf]
